# Phase I Study of GC1008 (Fresolimumab): A Human Anti-Transforming Growth Factor-Beta (TGFβ) Monoclonal Antibody in Patients with Advanced Malignant Melanoma or Renal Cell Carcinoma

**DOI:** 10.1371/journal.pone.0090353

**Published:** 2014-03-11

**Authors:** John C. Morris, Antoinette R. Tan, Thomas E. Olencki, Geoffrey I. Shapiro, Bruce J. Dezube, Michael Reiss, Frank J. Hsu, Jay A. Berzofsky, Donald P. Lawrence

**Affiliations:** 1 Vaccine Branch and Metabolism Branch, Center for Cancer Research, National Cancer Institute, Bethesda, Maryland, United States of America; 2 Department of Medicine, The Cancer Institute of New Jersey, New Brunswick, New Jersey, United States of America; 3 Department of Medicine, The Ohio State University, Columbus, Ohio, United States of America; 4 Department of Medicine, Dana-Farber Cancer Institute, Boston, Massachusetts, United States of America; 5 Department of Medicine, Beth Israel Deaconess Medical Center, Boston, Massachusetts, United States of America; 6 Genzyme Corporation, Cambridge, Massachusetts, United States of America; 7 Department of Medicine, Massachusetts General Hospital, Boston, Massachusetts, United States of America; University Clinic of Navarra, Spain

## Abstract

**Background:**

In advanced cancers, transforming growth factor-beta (TGFβ) promotes tumor growth and metastases and suppresses host antitumor immunity. GC1008 is a human anti-TGFβ monoclonal antibody that neutralizes all isoforms of TGFβ. Here, the safety and activity of GC1008 was evaluated in patients with advanced malignant melanoma and renal cell carcinoma.

**Methods:**

In this multi-center phase I trial, cohorts of patients with previously treated malignant melanoma or renal cell carcinoma received intravenous GC1008 at 0.1, 0.3, 1, 3, 10, or 15 mg/kg on days 0, 28, 42, and 56. Patients achieving at least stable disease were eligible to receive Extended Treatment consisting of 4 doses of GC1008 every 2 weeks for up to 2 additional courses. Pharmacokinetic and exploratory biomarker assessments were performed.

**Results:**

Twenty-nine patients, 28 with malignant melanoma and 1 with renal cell carcinoma, were enrolled and treated, 22 in the dose-escalation part and 7 in a safety cohort expansion. No dose-limiting toxicity was observed, and the maximum dose, 15 mg/kg, was determined to be safe. The development of reversible cutaneous keratoacanthomas/squamous-cell carcinomas (4 patients) and hyperkeratosis was the major adverse event observed. One malignant melanoma patient achieved a partial response, and six had stable disease with a median progression-free survival of 24 weeks for these 7 patients (range, 16.4–44.4 weeks).

**Conclusions:**

GC1008 had no dose-limiting toxicity up to 15 mg/kg. In patients with advanced malignant melanoma and renal cell carcinoma, multiple doses of GC1008 demonstrated acceptable safety and preliminary evidence of antitumor activity, warranting further studies of single agent and combination treatments.

**Trial Registration:**

Clinicaltrials.gov NCT00356460

## Introduction

Transforming growth factor-beta (TGFβ) is a pleiotropic cytokine that is a member of a superfamily of ligands that includes bone morphogenetic proteins and activins [1], [2]. Under normal conditions, TGFβ helps to maintain homeostasis and limit the growth of epithelial, endothelial, neuronal, and hematopoietic cell lineages through anti-proliferative and apoptotic responses. In addition, TGFβ exerts potent effects that influence immune function, differentiation, adhesion, extracellular matrix production, cell motility, angiogenesis, and cytokine production [3], [4].

Early in the transition of premalignant lesions into malignant neoplasms, TGFβ can suppress cell growth; however, in advanced cancers these effects are typically lost. Instead, TGFβ will directly promote tumor growth and metastases [2], [4], [5]. Chronic exposure of transformed mouse keratinocytes to TGFβ causes a change in morphology and engenders these cells with the ability to form spindle cell carcinomas when transplanted into mice [6]. TGFβ induces epithelial-to-mesenchymal transition, which is characterized by a morphological change to a spindle cell shape, down-regulation of E-cadherin and cytokeratin expression, loss of cell-cell junctions, remodeling of the cytoskeleton, and increased cell motility [2], [4], [7]. TGFβ-induced cellular changes have been described in many different tumor models and appear to be important for inducing cell migration and promoting metastases [7].

Through its paracrine functions, TGFβ promotes remodeling of the microenvironment to support tumor growth and facilitate metastases. Remodeling of the tumor stroma occurs through the induction of vascular endothelial growth factor (VEGF) and angiogenesis, dysregulated lymphangiogenesis, increased extracellular matrix deposition, and production of factors such as parathyroid hormone-related peptide (PTHrP) that increase osteoclastic activity [4], [8]–[10]. TGFβ also attenuates host antitumor immune responses. With broad activity in natural killer (NK) cells, T cells including T regulatory cells, NKT cells, monocytes/macrophages, and dendritic cells, TGFβ can down-regulate both primary and secondary immune responses and suppress antitumor effector cells [3], [11], [12].

Increased TGFβ expression has been reported in many different malignancies including prostate, breast, lung, pancreatic, renal cell, liver, colon, gastric, esophageal, ovarian, cervical, bladder, thyroid, head and neck cancers, melanoma, gliomas, and multiple myeloma [13], [14]. Furthermore, elevated plasma TGFβ levels correlate with advanced tumor stage, metastases, and poor survival [15]–[17]. Given its integral role in the progression of cancer, TGFβ is an attractive therapeutic target.

In a number of preclinical models, neutralizing antibodies or soluble receptors that bind TGFβ have demonstrated antitumor activity [10], [18]–[23]. In murine metastatic breast cancer models, a survival benefit and a reduction in the incidence and size of lytic bone lesions and lung metastases were observed in animals receiving anti-TGFβ antibody therapy alone [20], [24] as well as when combined with chemotherapy [25]. Similarly, in B16 murine melanoma, anti-TGFβ therapy alone [26] or in combination with interleukin-2 reduced the number of lung metastases [27]. Additive effects of anti-TGFβ combined with various chemotherapies, radiation or biologics including vaccines have been reported to improve the treatment of both primary and metastatic disease [21], [25], [27]–[30].

GC1008, or fresolimumab, is a high-affinity fully human monoclonal antibody that neutralizes the active form of human TGFβ1, β2, and β3. It was designed as an IgG_4_ isotype to minimize immune effector function. GC1008 is being investigated as a treatment for cancer and fibrotic diseases [31]. Herein, we report our results on the safety and antitumor activity of repeated doses of GC1008 administered to patients with advanced malignant melanoma (MM) and renal cell carcinoma (RCC).

## Materials and Methods

### Ethics Statement

The protocol was approved by the Institutional Review Board (IRB) at each participating site: the National Cancer Institute IRB; the Dana Farber-Harvard Cancer Center IRB (Office for the Protection of Research Subjects); the Cancer Institute of New Jersey (University of Medicine and Dentistry of New Jersey) IRB; and the Ohio State University IRB. In addition, after site IRB approval, the Ohio State University and the Cancer Institute of New Jersey utilized the services of the Western Institutional Review Board. Written informed consent was obtained from each patient. The trial was monitored by an independent Data Monitoring Committee (DMC). The protocol for this trial and supporting CONSORT checklist are available as supporting information; see Checklist S1 and Protocol S1

### Study design and patients

The primary objectives of this phase I trial (clinicaltrials.gov: NCT00356460) were to determine the maximum tolerated dose (MTD), dose-limiting toxicity (DLT), and safety of GC1008 in patients with MM or RCC. Secondary objectives were to obtain pharmacokinetic and pharmacodynamic data on GC1008, estimate clinical activity, and evaluate exploratory biomarkers. After completion of the initial Dose-Escalation phase (Part 1), the study was amended to enroll and treat additional patients with 15 mg/kg GC1008 (Part 2). The purpose of the expansion cohort was to acquire additional data and examine the relationship of GC1008 to certain observed skin lesions.

**Figure 1:**
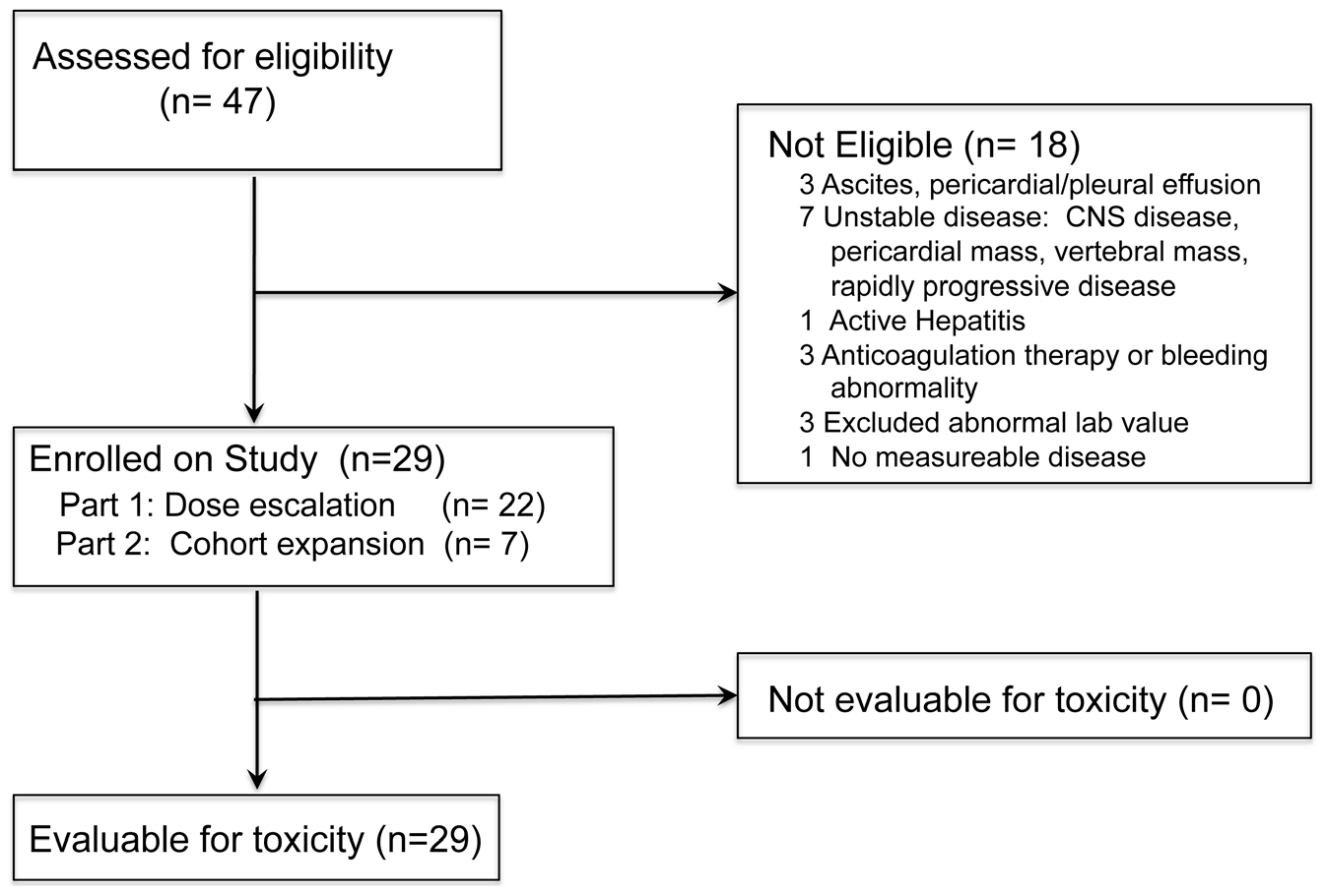
Patient Flow Chart.

Patients ≥18 years old with metastatic or non-resectable MM or RCC who had received at least one prior therapy were eligible. Subjects were >4 weeks since major surgery, radiotherapy, or prior systemic treatment, and prior treatment-related toxicity returned to ≤grade 1. RCC patients must have previously received and progressed on sorafenib or sunitinib, and temsirolimus after becoming available. Eastern Cooperative Oncology Group (ECOG) performance status ≤2; measurable disease; absolute granulocyte count ≥1,500/µL; hemoglobin ≥10.0 g/dL; platelets ≥100,000/µL; serum creatinine <2 mg/dL or creatinine clearance ≥60 mL/minute; total bilirubin ≤1.5× upper limit of normal (ULN); ALT or AST ≤2.5× ULN (≤5× ULN with liver metastases); negative tests for viral hepatitis and HIV; and an expected survival ≥5 months were required. Key exclusions were central nervous system (CNS) metastases; ascites or pleural effusion; active bleeding; another invasive malignancy within 5-years; autoimmune disease; immunosuppressive or anticoagulation medications, or uncontrolled unrelated physical or mental illness. Patient flow is shown in Figure 1.

### Treatment

**Figure 2:**
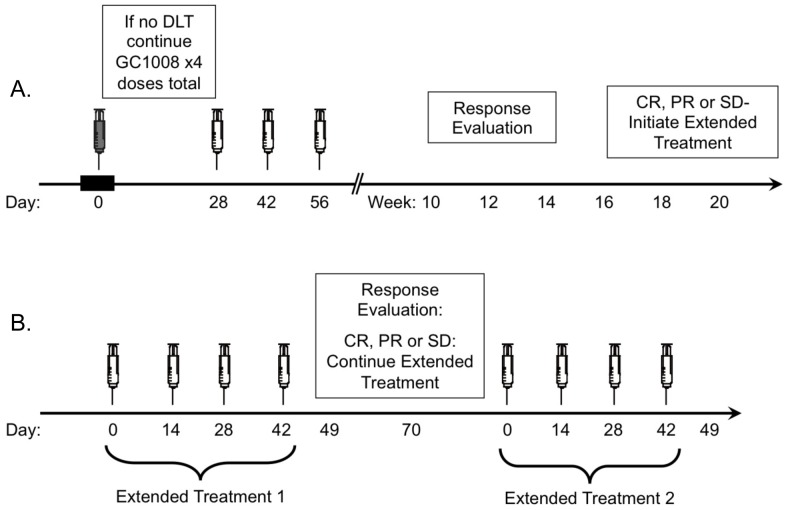
Trial Design. **A**. GC1008 treatment and dose-escalation schema. Groups of 3 to 6 subjects received an initial dose of GC1008 and were monitored for 28 days for DLT. Subjects experiencing no DLT received three additional doses of GC1008 every 2 weeks. Subjects underwent assessment of tumor response week 12 that was confirmed at least 4 weeks later. **B**. Extended Treatment schema. Subjects who achieved a response of stable disease or better were eligible for Extended Treatment at the current dose of GC1008 determined to be safe which was administered every 2 weeks ×4 doses. Up to two cycles of Extended Treatment could be given as long as the response was maintained.

During the Dose-Escalation phase (Part 1), sequential cohorts of 3 to 6 patients received intravenous infusions of GC1008 (fresolimumab, Genzyme Corporation, Cambridge, MA.) at doses of 0.1, 0.3, 1, 3, 10, or 15 mg/kg. Premedication with diphenhydramine and acetaminophen was suggested but not required. Patients received 3 additional infusions of GC1008 at the same dose on days 28, 42, and 56 (Figure 2A). Advancement of dose cohorts occurred if 0 of 3, or ≤1 of 6 patients experienced a DLT within 28 days of the first GC1008 dose. At the highest planned dose of 15 mg/kg, if ≤1 of 6 patients experienced a DLT, this was designated the maximal safe dose; if not, the MTD was defined as the highest dose-level in which ≤1 of 6 patients experienced a DLT.

Patients with confirmed responses of stable disease (SD) or better were eligible to receive up to 2 courses of Extended Treatment, each consisting of 4 doses of GC1008 administered every 2 weeks (Figure 2B). For each course, intrapatient dose escalation to the current GC1008 dose determined to be safe was allowed. After completion, patients were followed every 3 months for up to 2 years.

### Safety and efficacy

Toxicities were graded using the NCI Common Terminology Criteria (CTCAE) version 3.0, the current version at study initiation. DLT was defined as any ≥grade 3 adverse event (AE) at least possibly related to GC1008 occurring within 28 days of the first dose of GC1008 with the following exceptions: Grade 3/4 acute infusion reaction that resolved with therapy in ≤4 hours; neutropenia or thrombocytopenia resolving ≤96 hours; effectively treated diarrhea or vomiting; grade 2 AST, ALT or bilirubin elevation at baseline increasing ≤3× baseline and resolving ≤5 days; or <grade 4 fatigue. Complete blood count, PT/PTT, serum chemistries, and platelet aggregation assay or bleeding time were performed at predetermined intervals.

Response was assessed using RECIST criteria [32] evaluated by physical exam and imaging studies that included CT or MRI, radionuclide bone scan and positron emission tomography (PET). Tumor measurements were assessed at baseline and at days 84 and 140. Responses were confirmed ≥4 weeks after initial determination. Patients on Extended Treatment were re-evaluated for response 4 weeks after completion of the Extended Treatment course.

### GC1008 pharmacokinetics and exploratory biomarkers

GC1008 levels were measured by ELISA-based assay [31] from blood samples collected on days 0, 1, 3, 7, 14, 28, 42, 56, 63, 70, 84, 112, 140 and every three months during follow up. The presence of human anti-human antibodies directed against GC1008 was also assessed at these time points with an electrochemiluminescence detection assay utilizing GC1008 labeled with a sulfo-tag. Blood samples were obtained for analyses of exploratory biomarkers, including plasma TGFβ, VEGF, peripheral blood mononuclear cells (PBMC) phospho-Smad, and immune function markers using enzyme-linked immunosorbent assay (ELISA) and flow cytometry. Samples of the original tumor were examined for expression of TGFβ 1 and 2, TGFβ receptors 1 and 2 and other markers.

### Skin histopathology

Patients developing any new or suspicious skin lesions underwent a biopsy. All cases were reviewed by the local institution and also by an independent central review panel that included a dermatopathologist and dermatologist. Central review was initially performed in a blinded manner and then again after receiving the clinical history.

### Statistical analysis

All patients who signed informed consent and received at least one dose of GC1008 were included in the analysis of AEs and response. Analyses included the proportion of patients achieving complete response (CR), partial response (PR) or SD, duration of response, and progression-free survival (PFS).

## Results

### Patients

Twenty-nine patients (28 MM and 1 RCC) with a median age of 59.0 years (range, 44–84 years) were treated on study between October 2006 and August 2009 (Table 1 and Table S1). Twenty-two patients were treated in Part 1 (Dose-Escalation), and following a pause to evaluate emergent skin lesions, 7 additional patients were treated in Part 2 as a safety expansion cohort at a dose of 15 mg/kg. A majority of patients (17/29) received all 4 planned doses of GC1008 and 5 of these patients received Extended Treatment. Among the 29 patients, the median cumulative dose was 1,186 mg (range, 28–6,689 mg). One patient from Cohort 1 (0.1 mg/kg) and one patient from Cohort 2 (0.3 mg/kg) received 4 additional doses of GC1008 at 1 mg/kg; three patients from Cohort 3 (1 mg/kg) received 4 additional doses each at 3 mg/kg, and one of these patients also received 4 doses at 15 mg/kg on Extended Treatment. All patients were evaluable for toxicity and response.

**Table 1:** Patient Demographics.

		All Patients (N = 29)	
		n	%
**Sex**	Male	17	59
	Female	12	41
**Race/Ethnicity**	White, non-Hispanic	27	93
	African-American	1	3.5
	Hispanic	1	3.5
**Age at enrollment (yrs)**	30–50	6	21
	51–60	9	31
	>60	14	48
**Type of cancer**	Renal Cell Carcinoma	1	3
	Malignant Melanoma	28	97
**ECOG status:**	0	19	66
	1	9	31
	2	1	3
**Prior Surgery** (Diagnostic or therapeutic)		29	100
**Prior Non-Surgical Therapies**			
	Chemotherapy, Kinase inhibitors^1^	17	59
	Biologics^2^	21	72
	Investigational Vaccines	8	28
	Radiation	6	21

1 Kinase inhibitors included sorafenib, gamma secretase inhibitor, dasatinib.

2 Biologics: Many were investigational agents and included Interleukin-2, interleukin-12, interferon alpha, interferon gamma, FLT-3 ligand, anti-CD137 Ab and anti-CTLA4 Ab. Anti-CTLA4 Ab therapy has been associated with delayed responses. In this study, 3 patients had received prior anti-CTLA4 therapy with the last treatment administered 8 months to 2 years before GC1008.

### Safety

AEs considered possibly related to study treatment and occurring in >2 patients or reported as CTCAE ≥grade 3 are shown in Table 2. Observed AEs included gingival bleeding (4 patients), epistaxis (4 patients), headache (4 patients), fatigue (3 patients) and various skin disorders. These AEs were ≤grade 2 with the exception of four grade 3 skin events. Thirteen patients (45%) experienced at least one serious adverse event (SAE), and 2 of these patients (7%) experienced a drug-related SAE, a squamous cell carcinoma (SCC) of the skin and an episode of herpes zoster. No study drug-associated deaths occurred and no acute infusion reactions were reported. There were no drug-related grade 4 or 5 SAEs, and no AE led to study drug discontinuation. Twelve patients received less than 4 doses of GC1008 due to documented disease progression or disease-related death (1 case). Although TGFβ exhibits tumor suppressor activity in pre-malignant lesions, there was no evidence of accelerated tumor growth due to blocking its effects in these advanced cancers, although this would be difficult to detect in a phase I study. Two patients, both receiving 15 mg/kg, died within 45 days of the last dose of GC1008. Both deaths were assessed as not related to study drug; one death was due to disease progression and the other was due to aspiration pneumonia

**Table 2:** Summary of Adverse Events Related to Study Treatment That Occurred in >2 Patients or Reported As CTCAE Grade 3 or Higher.

	Total (N = 29)				
	Grade 1	Grade 2	Grade 3	Grade 4	Any Grade
Toxicity^1^	n (%)	n (%)	n (%)	n (%)	n (%)
Any	10 (34.5)	4 (13.8)	4 (13.8)	0	18 (62.0)
Gingival bleeding	4 (13.8)	0	0	0	4 (13.8)
Fatigue	3 (10.3)	0	0	0	3 (10.3)
Headache	2 (6.9)	2 (6.9)	0	0	4 (13.8)
Epistaxis	4 (13.8)	0	0	0	4 (13.8)
Herpes zoster^2^			1		
Skin Disorders					
Actinic keratosis	1 (3.4)	1 (3.4)	1 (3.4)	0	3 (10.3)
Hyperkeratosis	1 (3.4)	2 (6.9)	0	0	3 (10.3)
Lichenoid keratosis	2 (6.9)	1 (3.4)	0	0	3 (10.3)
Rash papular	2 (6.9)	1 (3.4)	0	0	3 (10.3)
Neoplasms, Benign and Malignant^3^					
Basal cell carcinoma	0	0	1 (3.4)	0	1 (3.4)
Keratoacanthoma	0	1 (3.4)	1 (3.4)	0	2 (6.9)
Squamous cell carcinoma of skin	0	0	2 (6.9)	0	2 (6.9)

1 If a patient had more than one event for a particular adverse event term, he/she is counted only once for that term and patient percentages are based on the total number of treated patients in the particular treatment group. There were no deaths attributed to study drug.

2 Herpes zoster was reported in two additional patients (both grade 2) but events were considered unrelated to GC1008. All 3 cases were treated with oral anti-viral agents and resolved within 1–2 weeks of diagnosis.

3 In CTCAE version 3, non life-threatening treatment related secondary malignancies are considered grade 3. Note: CTCAE version 4 added a new category “Neoplasms benign, malignant and unspecified” which corrected a lack of grade 1 and 2 designations. Under version 4, some of these lesions may have been considered grade 2. On review, the 2 cases diagnosed at the treating site as SCC were interpreted by the independent central review as representing KAs, and in one case involving a patient with prior SCC, an atypical squamous-epithelial-proliferation with KA-like features was read as most consistent with SCC.

In preclinical toxicology studies, a dose- and duration-dependent anemia was noted in a small number of test animals that was reversible and attributed to bleeding oral mucosal hyperplasia. In patients, similar lesions were not observed. A small number of minor clinical bleeding events were reported, but their relationship to GC1008 is unclear. No study drug-related abnormalities were observed in platelet number or function or coagulation parameters that might indicate a bleeding diathesis. Three cases of herpes zoster were observed at different time points following completion of therapy (12, 44, and 45 days from last dose and 75, 100, and 340 days from first dose). One patient completed 12 doses of GC1008 before the outbreak of zoster; another received 4 doses afterwards without recurrence. The DMC concluded that there was insufficient evidence of a causal relationship of GC1008 to herpes zoster.

Skin toxicity was the most common drug-related AE/SAE observed and, per local site pathological review, included the development of eruptive keratoacanthomas (KA) (Figure S1) and hyperkeratosis (2 patients), cutaneous SCC (2 patients), and basal cell carcinoma (1 patient) (see Table 2). In 3 patients, transient non-specific papular rash or hyperkeratotic lesions preceded the development of KAs or SCC lesions. KAs occurred in a patient who received treatment at 1 mg/kg and then two courses of Extended Treatment at 3 and 15 mg/kg and in another patient who received initial treatment at 15 mg/kg. Study site reported cases of SCC occurred in a patient who received treatment at 1 mg/kg and then Extended Treatment at 3 mg/kg and in another patient who received initial treatment at 15 mg/kg. In each KA/SCC case, the pathological diagnoses were made after completion of all GC1008 doses (3 patients) or during treatment at their highest dose level (1 patient who received 2 courses of Extended Treatment), indicating a possible association of dose exposure and development of these lesions. Patients underwent local treatment, and following completion of GC1008, remaining lesions regressed over a period of weeks to months, supporting a benign, non-malignant, clinical behavior and the diagnosis of KA.

Twenty-four skin biopsies were sent for an independent, blinded pathological analysis. On review, the 2 cases diagnosed at the treating site as SCC were interpreted by the independent central review as representing KAs, and in one case involving a patient with prior SCC, an atypical squamous-epithelial-proliferation with KA-like features was read as most consistent with SCC. A variety of other grade 1 or 2 related and unrelated skin rashes were reported in 10 patients that improved or resolved by end of study. Six patients continued therapy without worsening of symptoms and none discontinued due to skin events. The KAs fully resolved over several weeks to months after discontinuation of GC1008, confirming that these were not autonomous malignancies.

No DLT was observed in Part 1, Dose-Escalation. Therefore, 15 mg/kg, the highest dose of GC1008 examined in this study, was determined to be the maximal safe dose.

### Response

**Figure 3:**
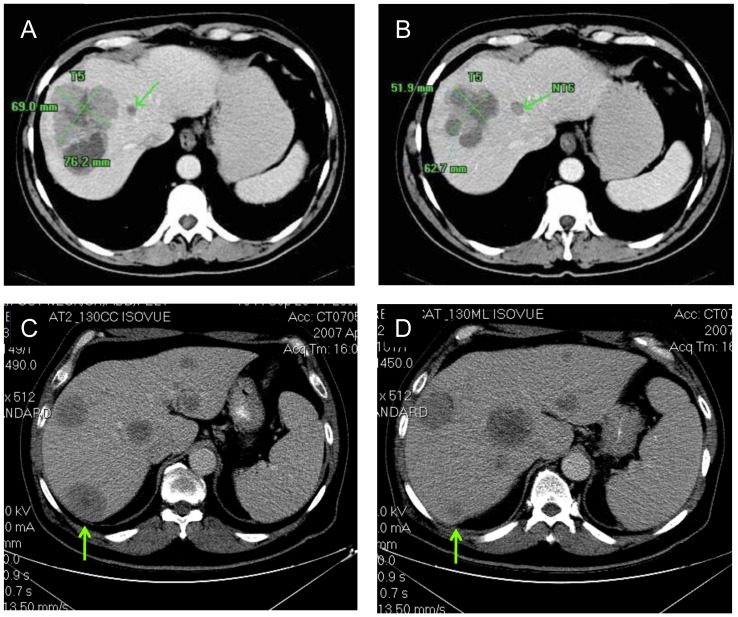
Mixed-tumor responses in patients with metastatic melanoma receiving GC1008. Patient 003 was previously treated with dacarbazine and sorafenib and then received GC1008 0.1/kg ×4 doses followed by 1 mg/kg ×4 doses on Extended Treatment. Representative CT scans comparing baseline (Panel A) and day 112 (2 months post Extended Treatment, Panel B) demonstrate a reduction in size of a liver metastasis (arrows) with features consistent with increased necrosis and cyst formation; however, a pulmonary hilar lesion demonstrated growth (not shown). Patient 008 was previously treated with multiple surgeries, high dose interleukin-2, and an investigational anti-melanoma vaccine and then received GC1008 1 mg/kg ×4 doses followed by 3 mg/kg ×4 doses on Extended Treatment. Comparison of CT scans from baseline (Panel C) and day 91 (Panel D) demonstrate near resolution of a liver metastasis (arrows); however, growth was noted in other hepatic lesions.

**Figure 4:**
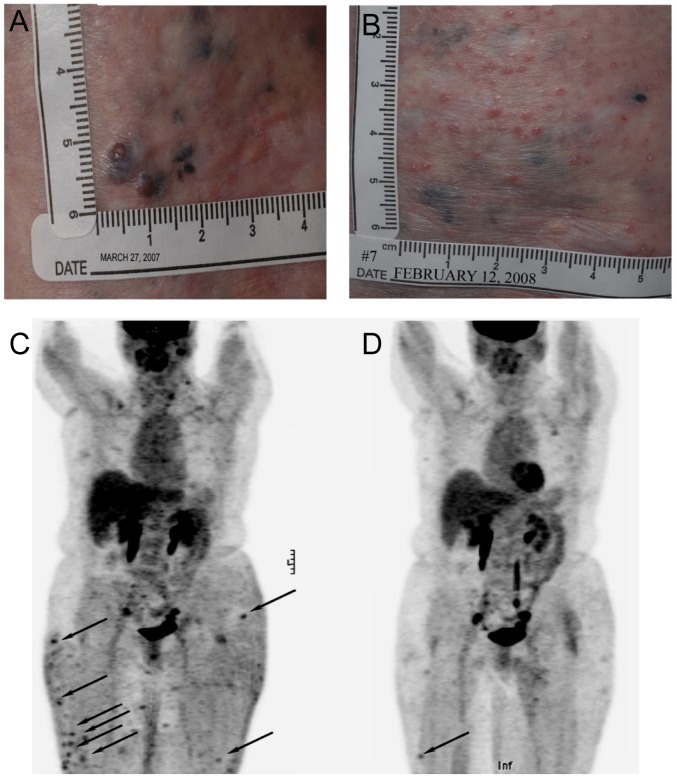
Partial response in Patient 007 with metastatic melanoma receiving GC1008. Patient was previously treated with multiple surgical resections, adjuvant interferon-alpha, interleukin-2, combination chemotherapy, hyperthermia, investigational anti-melanoma vaccines, and ipilimumab and then progressed and presented with extensive cutaneous and subcutaneous metastatic disease. The patient received GC1008 initially at 1 mg/kg ×4 doses followed by 2 courses of Extended Treatment at 3 mg/kg ×4 doses and 15 mg/kg ×4 doses and achieved a partial response with 89.6% decrease in size of her target lesions. Reduction of cutaneous lesions were noted by serial measurements (Panel A baseline, Panel B day 308) and by PET scans (Panel C baseline, Panel D day 218 following 8 doses).

Of the 29 patients, 1 MM patient achieved a PR, and 6 MM patients experienced SD including 3 patients with mixed tumor responses (Table 3, Figures 3 and 4). The median PFS for all 29 patients was 11.1 weeks (range, 4.1–44.4 weeks). The median time to progression (TTP)/PFS for the PR and SD patients was 24 weeks (range, 16.4–44.4 weeks) (Table 3). The responding patient had extensive cutaneous and subcutaneous disease and achieved a partial response with 89.6% reduction in target lesion size lasting 44.4 weeks (Figure 4 and Figure S2). SD or better occurred at initial GC1008 doses of 0.1, 0.3, 1, 3 and 15 mg/kg GC1008; although most initially received ≤3 mg/kg GC1008, 5 patients also received 1 or 2 courses of Extended Treatment with intrapatient dose escalation to 1, 3, or 15 mg/kg GC1008 (Table 3).

**Table 3:** Patient Responses.

Patient/Disease	Dose Cohort (mg/kg)	Extended Treatment	Response	Comment	Progression-free survival (weeks)
003 Melanoma	0.1	4 doses of 1 mg/kg	Stable disease	Mixed response: Liver and pulmonary lesions and lymph nodes	31.1
006 Melanoma	0.3	4 doses of 1 mg/kg	Stable disease	Liver lesions decreased 9.2% in size. Patient reported improvement in breathing and less fatigue.	22
007	1	4 doses at 3 mg/kg	Partial response: 89.6% decrease in	PET-CT: Skin lesions improved	44.4
Melanoma		4 doses at 15 mg/kg	skin lesions		
008 Melanoma	1	4 doses at 3 mg/kg	Stable disease	Mixed response: Liver lesions	24
009 Melanoma	1	4 doses at 3 mg/kg	Stable disease		27.1
012 Ocular melanoma	3	None	Stable disease		16.4
018 Melanoma	15	None	Stable disease		20.1

Median time to progression/progression-free survival, 24 weeks (range, 16.4 to 44.4 weeks).

#### GC1008 pharmacokinetics and exploratory biomarkers

**Figure 5:**
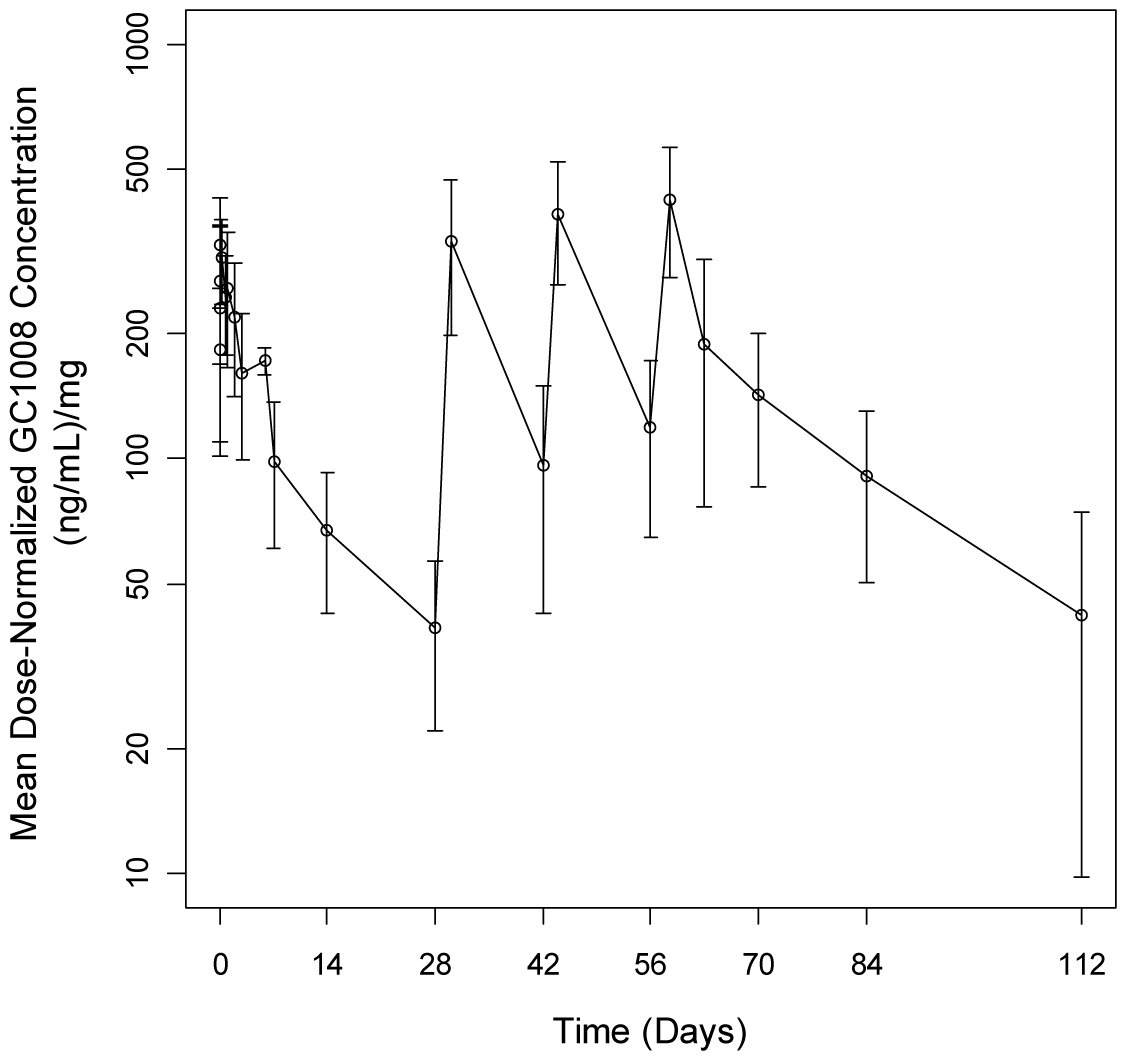
Pharmacokinetics. GC1008 levels were measured from blood samples collected on days 0, 1, 3, 7, 14, 28, 42, 56, 63, 70, 84, 112, 140 and every three months during follow up. Mean dose-normalized GC1008 concentrations (mean ±standard deviation) were determined. Pharmacokinetic data for all patients through the first 4 doses of treatment are represented in this plot. Concentrations are normalized to the administered dose, and no intrapatient dose escalations are included. Accumulation of the antibody was ≤2.5-fold at the C_min_ and <1.6-fold at C_max_. When all pharmacokinetic data is examined, including that from patients receiving Extended Treatment, GC1008 demonstrated an overall calculated half-life of 21.7 days.

GC1008 pharmacokinetics were determined to be linear and dose proportional. GC1008 demonstrated an overall calculated half-life of 21.7 days. Accumulation of the antibody was ≤2.5-fold at the C_min_ and <1.6-fold at C_max_. Dose-normalized pharmacokinetics are shown in Figure 5. Pre- and post-treatment serum samples were examined for anti-GC1008 antibodies. One positive titer was observed in a patient following GC1008 treatment and was detected at 1∶30, the lower limit of quantitation for the assay. Interpretation of anti-GC1008 antibody results should be made with caution since the presence of circulating GC1008 can interfere with the assay. The clinical significance of this low titer anti-GC1008 antibody is unclear.

**Figure 6:**
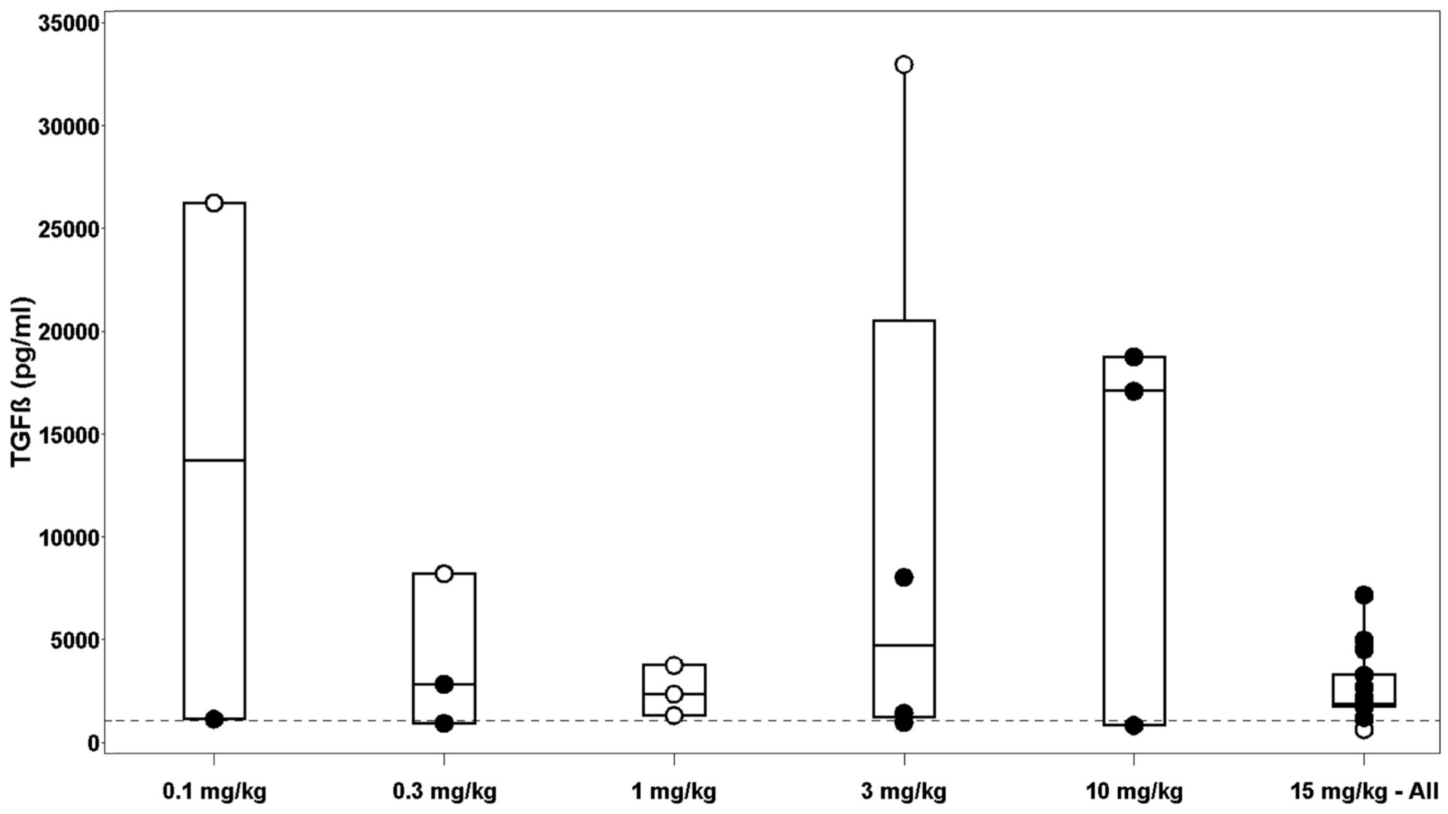
Pretreatment TGFβ plasma levels. Measurements of total TGFβ levels were performed on plasma using an ELISA assay. Boxplots of baseline levels of TGFβ are displayed for each patient by cohort. The dashed line represents the normal range, as determined by 42 healthy donors with a mean ±standard deviation of 1051±221 pg/mL. Open circles indicated baseline levels of patients who achieved a PR or SD.

Several exploratory biomarkers were examined in this trial. Plasma TGFβ levels at baseline were elevated in the majority of patients, including those experiencing SD or better (Figure 6). Due to the small sample size of this study, the ability to interpret and correlate TGFβ levels and clinical outcome was limited. Expression levels of other exploratory biomarkers including plasma VEGF, PBMC phospho-Smad, tumor cell TGFβ and its receptors were examined, but could not be correlated to tumor characteristics or clinical outcome. However, these results should be considered preliminary due to the small sample size. Further evaluation of these exploratory biomarkers is planned in future studies.

## Discussion

TGFβ is a pleiotropic cytokine with complex biology that affects tumor, its microenvironment, and the tumor's ability to evade the immune system. In many advanced cancers, TGFβ plays a critical role in tumor growth, disease progression and the development of metastases. Therefore, targeting and neutralizing TGFβ would be predicted to have therapeutic benefit. Importantly, even though blockade of TGFβ may not be directly tumoricidal, it affects key elements of local tumor growth, host defenses and the ability to metastasize, all of which may be additive or synergistic when combined with other anti-cancer therapies.

In this phase I study, GC1008, a human monoclonal antibody that neutralizes all three human isoforms of TGFβ, was examined. GC1008 demonstrated an acceptable safety profile. No DLTs were observed and 15 mg/kg, the highest dose investigated, was determined to be safe. GC1008 demonstrated linear and dose-proportional pharmacokinetics and had an overall calculated half-life of 21.7 days. Preliminary evidence of clinical benefit was observed in 7 patients who achieved either a PR (1 patient, 89.6% reduction in tumor measurements) or SD (6 patients, including 3 with mixed tumor response) and had a median TTP/PFS of 24 weeks (range, 16.4–44.4 weeks). Although the number of patients was small, the median PFS for all 29 patients was 11.1 weeks, which was comparable to the median TTP/PFS of 1.3 to 2 months reported for patients with advanced MM treated with dacarbazine or other chemotherapy [33], [34].

Notable safety observations included a variety of treatment-emergent skin lesions including hyperkeratosis and eruptive KA/SCCs. KAs are epidermal tumors characterized by initially rapid growth typically followed by spontaneous resolution [35]. KAs can be a diagnostic challenge and are difficult to distinguish from well-differentiated cutaneous SCCs [35], [36]. KA/SCCs developed in 4 patients as multiple waxing and waning lesions (Figure S1). The development of KA/SCCs and cutaneous hyperkeratosis appeared to be associated with greater GC1008 exposure, occurring in patients treated at higher doses, those receiving Extended Treatment, or both.

Suspicious skin lesions were followed with sequential biopsies. Independent blinded central pathology review found that all but one lesion in a single patient were consistent with KAs. Most patients developed multiple lesions, many of which were not removed and were carefully observed after GC1008 treatment was temporarily interrupted or after study completion. Importantly, these lesions spontaneously resolved over a period of weeks to months, a finding consistent with the diagnosis of non-malignant KAs and not of true SCC.

KAs may represent a biological and pharmacodynamic effect of TGFβ neutralization. Ferguson-Smith disease is an autosomal-dominant skin disorder characterized by multiple, locally invasive SCC–like skin tumors that grow rapidly for a few weeks before spontaneously regressing [35]. A recent genomic analysis of patients with this disease indicated a correlation between loss-of-function mutations of the TGFβR1 receptor and the development of KAs [37]. The lesions observed in patients receiving GC1008 are similar to those described in this disorder. Therefore, the development of KAs is suggestive of an on-target effect of GC1008 where neutralization of TGFβ by GC1008 appears to mimic the loss of TGFβ signaling found in patients with Ferguson-Smith disease and is consistent with a clinical TGFβ neutralizing effect of GC1008. Interestingly, KAs and SCCs have been reported in patients receiving sorafenib, B-RAF inhibitors, and anti-tumor necrosis factor (TNF) α-directed therapies [33], [38], [39], and recent data with vemurafemib have implicated RAS pathway mutations and signaling through the mitogen-activated protein kinase pathway in the development of these lesions [40]. The exact link between these drugs and the effects of GC1008 are unknown.

In this study, 6 of 7 patients achieving a PR or SD were initially assigned to receive doses of GC1008 of 3 mg/kg or less. This may suggest an inverse dose-effect with improved responses observed at lower doses. However, bias in this small sample of patients cannot be ruled out and 5 of these patients received Extended Treatment with intrapatient dose escalation to higher levels than their initial treatment dose. A phase II study comparing high and low doses of GC1008 is needed to verify or disprove this impression. The impact of higher doses on clinical outcome was also explored. The median PFS observed in the combined 10 and 15 mg/kg cohorts was 7.2 weeks (range, 4.1–20.1 weeks, mean 8.9 weeks), which is consistent with the median TTP/PFS of 1.3 to 2 months reported for patients with MM treated with dacarbazine or other chemotherapy [33], [34]. Based on this, there appears to be no negative impact of higher doses of GC1008 on clinical outcome. Moreover, the limited data on PFS (Table 3) suggests that some patients may have benefited beyond what would be expected from such standard-of-care therapy. This is notable in that the majority of anti-TGFβ activity may be to suppress tumor growth and metastases through its effects on epithelial-to-mesenchymal transition, immune response and tumor microenvironment, rather than an ability to directly kill tumor cells.

Future studies will examine combination therapy. A variety of anti-TGFβ agents combined with chemotherapy, radiation, biologics, immunotherapeutics and vaccines have demonstrated enhanced antitumor effects in preclinical models [11], [21], [22], [25], [28]–[30], [41]–[44]. Several mechanisms have been proposed to explain the improved efficacy with cytotoxic agents and include increased diffusion of chemotherapy and payload-containing particles such as liposomes into tumor masses [45], [46], reversal of chemotherapy resistance [29], [41], inhibition of DNA repair by blockade of γH2AX foci formation [28] and reversal of TGFβ-induced accumulation of tumor cells in G_0_/G_1_ [21], [44]. In addition, novel anti-TGFβ activity such as inhibition of angiogenesis, fibrosis, metastases, and immune suppression may be additive to other treatments [4], [10], [30]. The potential to inhibit suppressive immune cells, cytokines and enzymes was recognized by the National Cancer Institute (NCI) Immune Response Modifier Pathway Prioritization Working Group, who recommended anti-TGFβ agents be investigated for their ability to augment immune responses to vaccines and cell therapy [47].

TGFβ is a central mediator of epithelial-to-mesenchymal transition, cell migration and metastasis. Control of these effects may be critical in the development of effective anti-cancer therapies. Norton and Massague [48], [49] have proposed that cancer cell migration and “re-seeding” of the primary tumor with cells primed for metastatic growth leads to a tumor with heterogenous subpopulations of aggressive, rapidly growing cancer cells that may become resistant to treatment. If correct, new anti-metastatic agents like GC1008 may prevent tumor re-seeding and the formation of aggressive cancers.

Work to better understand which patients might benefit from anti-TGFβ therapy is ongoing. Exploratory biomarkers such as pre-treatment TGFβ levels may prove to be useful in stratifying patients who may respond to GC1008. In this study, however, evaluation of these markers was limited by small patient numbers, and final conclusions will have to await larger studies. Gene expression analyses of metastatic and aggressive cancers have identified elevated expression of TGFβ-associated genes, especially those involved with epithelial-to-mesenchymal transition [50]. Significant overlap of the TGFβ response gene signature, lung metastasis signature, and basal cell breast subtype exist [50]. Recently, targeted therapies directed against B-RAF and other genes have been used successfully to treat patients with MM [33]. Studies of these genes and associated mutations were under investigation and no results were available when this trial of GC1008 was initiated. Therefore, analyses of tumor samples for these defects were not performed in this trial. In the future, identification of cancers with these gene signatures, specific B-RAF mutations, or biomarkers may facilitate the selection of cancers and patients that would benefit from this therapy.

In conclusion, GC1008 exhibited an acceptable safety profile when administered up to 15 mg/kg every 2 weeks. This profile and the preliminary evidence of antitumor activity indicate that additional studies are warranted. New studies have been initiated in mesothelioma, myelofibrosis and breast cancer. These and future studies will help characterize the efficacy and safety of single agent and combination approaches, as well as define dose and response. Importantly, the safety profile of GC1008 appears to be non-overlapping with traditional anti-cancer agents, a feature which should facilitate its development in combination regimens.

## Supporting Information

Checklist S1 **CONSORT checklist.**
(DOC)

Figure S1 **Skin Lesions.** Raised papules with a central keratin core appearing over the arm and hand of a patient treated with GC1008. Biopsies were consistent with keratoacanthomas.
(TIF)

Figure S2 **Non-target melanoma lesion from a responding patient.** Biopsy from patient 007 revealed tumor central necrosis and a lymphocytic infiltrate on hematoxylin-eosin stain. Additional immunohistochemical stains (not shown) demonstrated 1 to 2+ (5–50%) CD8+ T cells at the periphery of the tumor.
(TIF)

Protocol S1 **Trial protocol.**
(PDF)

Table S1 **Individual Patient Data.**
(DOC)
