# Supplementary figures and images for: Phase I Study of GC1008 (Fresolimumab): A Human Anti-Transforming Growth Factor-Beta (TGFβ) Monoclonal Antibody in Patients with Advanced Malignant Melanoma or Renal Cell Carcinoma
Source: PLoS One. 2014 Mar 11;9(3):e90353. doi: 10.1371/journal.pone.0090353 (PMC3949712; doi:10.1371/journal.pone.0090353)

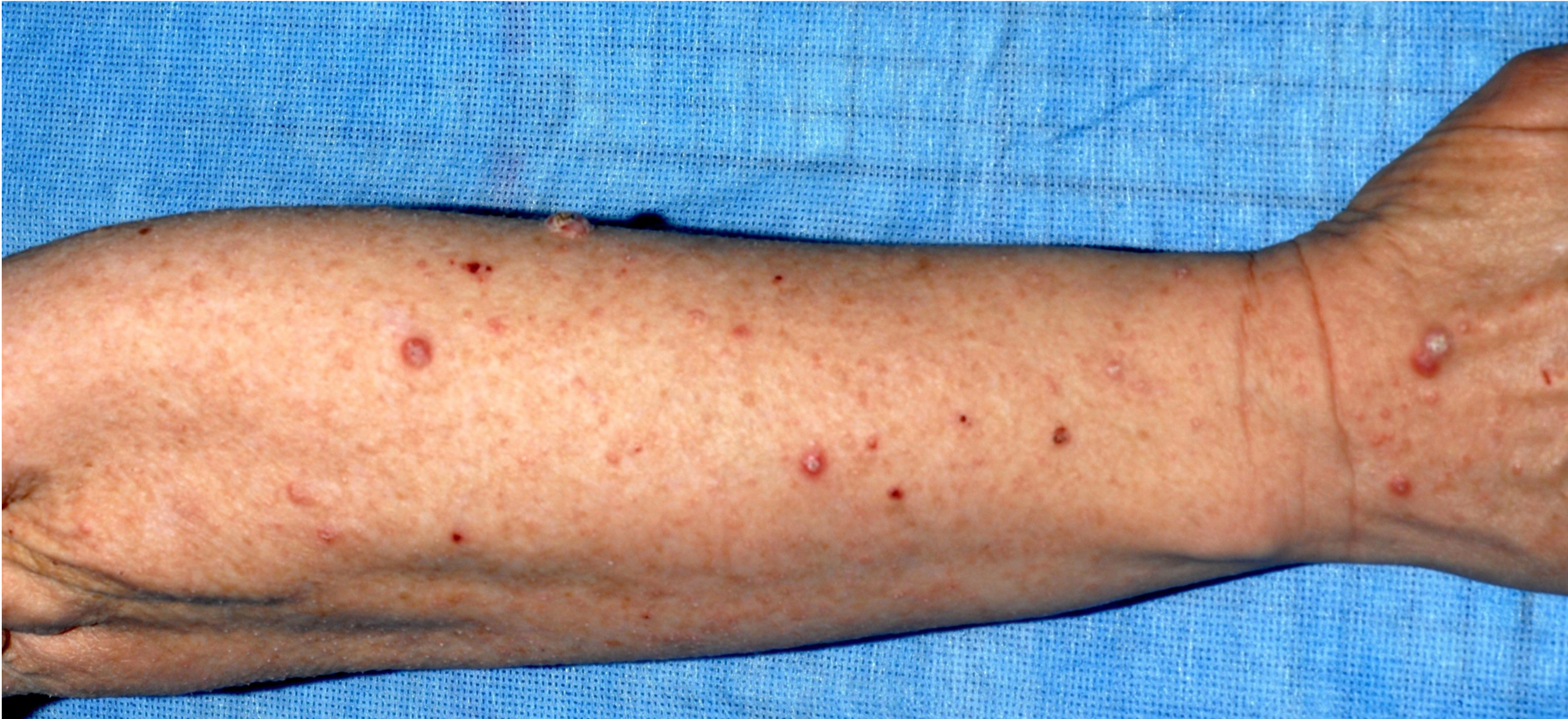

Supplement: Figure S1 — Skin Lesions. Raised papules with a central keratin core appearing over the arm and hand of a patient treated with GC1008. Biopsies were consistent with keratoacanthomas. (TIF) [file pone.0090353.s002.tif]

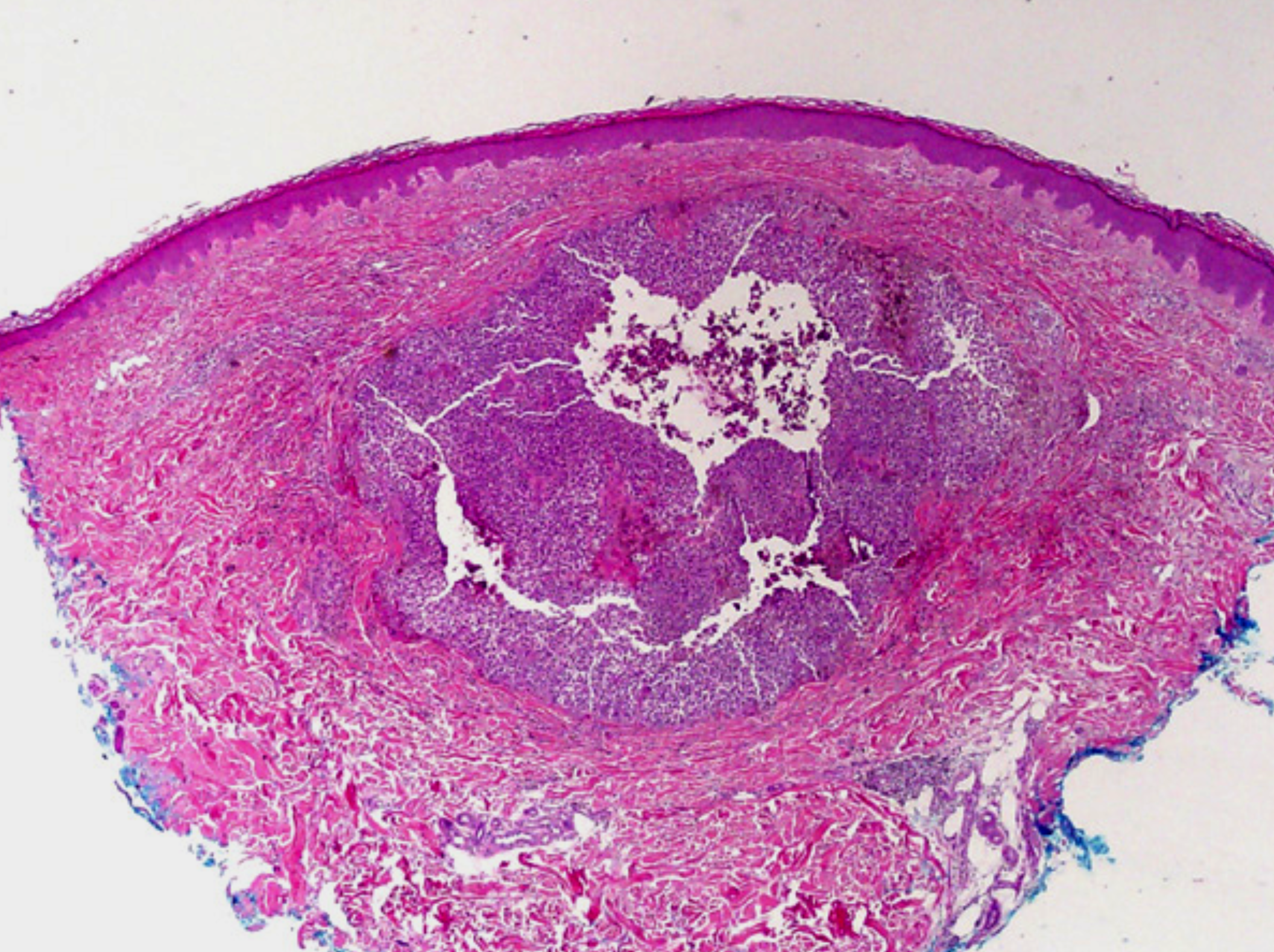

Supplement: Figure S2 — Non-target melanoma lesion from a responding patient. Biopsy from patient 007 revealed tumor central necrosis and a lymphocytic infiltrate on hematoxylin-eosin stain. Additional immunohistochemical stains (not shown) demonstrated 1 to 2+ (5–50%) CD8+ T cells at the periphery of the tumor. (TIF) [file pone.0090353.s003.tif]
