# Supplementary material for: Phase I Study of GC1008 (Fresolimumab): A Human Anti-Transforming Growth Factor-Beta (TGFβ) Monoclonal Antibody in Patients with Advanced Malignant Melanoma or Renal Cell Carcinoma
Source: PLoS One. 2014 Mar 11;9(3):e90353. doi: 10.1371/journal.pone.0090353 (PMC3949712; doi:10.1371/journal.pone.0090353)
Supplement: Protocol S1 — Trial protocol. (PDF) [file pone.0090353.s004.pdf]

PHASE 1 CLINICAL STUDY PROTOCOL

A Phase 1 Study of the Safety and Efficacy of GC1008: A Human Anti Transforming Growth Factor-beta (TGFβ) Monoclonal Antibody in Patients with Advanced Renal Cell Carcinoma or Malignant Melanoma

Amendment 6: 09 November 2009
Amendment 5: 30 June 2008
Amendment 4: 19 September 2007 (Withdrawn)
Amendment 3: 31 May 2007
Amendment 2: 12 January 2007
Amendment 1: 13 April 2006
Original Protocol: 17 February 2006

This protocol was designed and will be conducted, recorded, and reported in compliance with the principles of Good Clinical Practice (GCP) guidelines. These guidelines are stated in U.S. federal regulations as well as "Guidance for Good Clinical Practice," International Conference on Harmonization of Technical Requirements for Registration of Pharmaceuticals for Human Use.
I have read and agree to abide by the requirements of this protocol.

Investigator Signature	Date

1. TABLE OF CONTENTS

Section **Page**

1. TABLE OF CONTENTS 2
2. LIST OF ABBREVIATIONS AND TERMS 4
3. STUDY OBJECTIVES 6
3.1 Part 1, Dose Escalation 6
3.1.1 Primary Objective 6
3.1.2 Secondary Objectives 6
3.2 Part 2, Patient Expansion 6
3.2.1 Primary Objective 6
3.2.2 Secondary Objectives 6
4. INVESTIGATIONAL PLAN 7
4.1 Study Design 7
4.1.1 Part 1, Dose Escalation 7
4.1.2 Part 2, Patient Expansion 8
4.2 Patient Assignment and Identification 11
5. PATIENT POPULATION AND SELECTION 12
5.1 Inclusion Criteria 12
5.2 Exclusion Criteria 13
5.3 Patient Withdrawal 15
6. CLINICAL TRIAL MATERIAL ADMINISTRATION 17
6.1 Treatments Administered 17
6.1.1 Dosage 19
6.2 Investigational Product 19
6.2.1 Dose-Escalation Rules (Part 1) 20
6.2.2 Guidelines for Treatment Delay, Dose Reduction, and Discontinuation (Part 2) 20
6.2.2.1 Treatment Delays (Part 2 Only) 21
6.2.2.2 Dose Reduction (Part 2 Only) 21
6.2.2.3 Discontinuation of Treatment Due to Skin Events (Part 2 Only) 21
7. EFFICACY AND SAFETY VARIABLES 23
7.1 Efficacy and Safety Measurements Assessed and Study Flowchart 23
7.2 Adverse Events 31
7.2.1 Part 1: Dose-Limiting Toxicity 31
7.2.2 Non-Tolerable Skin Events 32
8. STATISTICAL METHODS AND PLANNED ANALYSES 33
8.1 Demographics and Baseline Characteristics 33
8.2 Concomitant Medication Usage 33
8.3 Patient Accountability 33

8.4 Study Treatment Usage and Compliance ... 33
8.5 Efficacy Endpoints ... 33
8.5.1 Intent-to-Treat (ITT) Population ... 34
8.5.2 Evaluable-for-Response Population ... 34
8.5.3 Safety Population ... 34
8.6 Safety Endpoints ... 34
8.6.1 Adverse Events ... 35
8.6.2 Laboratory Parameters ... 36
8.6.3 Other Safety Assessments ... 36
8.7 Maximum Tolerated Dose or Maximum Safe Dose ... 36
8.8 Pharmacokinetic Endpoints ... 36
8.9 Anti-GC1008 Antibody Analysis ... 37
8.10 Statistical Analysis Plan ... 37
8.11 Power and Sample Size ... 37
8.12 Other Statistical Issues ... 38
8.12.1 Significance Levels ... 38
8.12.2 Missing or Invalid Data ... 38
8.12.3 Computing Environment ... 38
9. REFERENCES ... 39

2. LIST OF ABBREVIATIONS AND TERMS

ABW	Actual body weight
AE	Adverse event
Akt	Agammaglobulinemia tyrosine kinase
ALT	Alanine aminotransferase (SGPT)
ANC	Absolute neutrophil count
AR	Androgen receptor
AST	Aspartate aminotransferase (SGOT)
BCC	Basal cell carcinoma
BUN	Blood urea nitrogen
CAT	Cambridge Antibody Technology
CBC	Complete blood count
CDDP	Cisplatinum
CHF	Congestive heart failure
CI	Confidence interval
CIN	Cervical intraepithelial neoplasia
CNS	Central nervous system
CR	Complete response
CRF	Case report form
CSSF	Clinical Supply Shipment Form
CT	Computerized tomography
CTCAE	Common Terminology Criteria for Adverse Events
CTX	Cyclophosphamide
DLT	Dose-limiting toxicity
DMC	Data Monitoring Committee
EC	Ethics Committee
ECG	Electrocardiogram
ECOG	Eastern Cooperative Oncology Group
eCRF	Electronic case report form
EDC	Electronic data capture
EGFR	Epidermal growth factor receptor
EMT	Epithelial mesenchymal transition
ER	Estrogen receptor
ERK	Extracellular signal-regulated kinase
GCP	Good Clinical Practice
GTPase	Guanosine triphosphatase
HIV	Human immunodeficiency virus
HuMab	Human monoclonal antibody
ICH	International Conference on Harmonisation
IgG	Immunoglobulin G
IL	Interleukin
IRB	Institutional Review Board
ITT	Intent-to-Treat

3. LIST OF ABBREVIATIONS AND TERMS (continued)

IV	Intravenous, intravenously
JNK	C-Jun N-terminal protein kinase
Kd	Dissociation constant
LAP	Latency-associated protein
LDH	Lactate dehydrogenase
Mad	Mothers against decapentaplegic (a Drosophila gene)
MAPK	Mitogen-activated protein kinase
MTD	Maximum tolerated dose
MRI	Magnetic resonance imaging
NCI	National Cancer Institute
NHP	Non-human primate
NK	Natural killer
NKT	Natural killer T-cell
NOAEL	No-observed-adverse-effect level
PAC	Paclitaxel
PAI-1	Plasminogen activator inhibitor-1
PD	Pharmacodynamic
PI3K	Phosphoinositide 3-kinase
PK	Pharmacokinetic
PO	Orally
PR	Partial response
PSA	Prostate-specific antigen
PT	Prothrombin time
PTHrP	Parathyroid hormone-related peptide
PTT	Partial thromboplastin time
QA	Quality assurance
RBC	Red blood cell
RECIST	Response Evaluation Criteria in Solid Tumors
SAE	Serious adverse event
sCr	Serum creatinine
SD	Stable disease
Sma	Small body size (a gene from Caenorhabditis elegans)
SMAD	Sma- and Mad-related proteins
TGFβ	Transforming growth factor-beta
TGFβRI, TGFβRII, TGFβRIII	Transforming growth factor-beta receptor type I, type II, type III
ULN	Upper limit of normal
VEGF	Vascular endothelial growth factor
WBC	White blood cell

3. STUDY OBJECTIVES

3.1 Part 1, Dose Escalation

3.1.1 Primary Objective

- To assess the maximum tolerated dose (MTD), dose-limiting toxicity (DLT), and safety of GC1008 in patients with locally advanced or metastatic renal cell carcinoma or malignant melanoma.

3.1.2 Secondary Objectives

- To obtain pharmacokinetic (PK) and pharmacodynamic (PD) data on GC1008.
- To evaluate tumor response as a preliminary assessment of clinical activity.

3.2 Part 2, Patient Expansion

3.2.1 Primary Objective

- To assess the safety of GC1008 following multiple doses at 15 mg/kg (or 10 mg/kg depending on the safety review of the first cohort of 6 patients at 15 mg/kg) in patients with locally advanced or metastatic malignant melanoma.

3.2.2 Secondary Objectives

- To obtain PK and PD data on GC1008.
- To evaluate tumor response as a preliminary assessment of clinical activity.
- To evaluate the relationship between GC1008 exposure, clinical response, and the development of skin lesions

4. INVESTIGATIONAL PLAN

4.1 Study Design

This is a Phase 1 multi-center, open-label, dose-escalation study designed to characterize the safety, tolerability, PK, PD, and potential anti-tumor activity of GC1008 in patients with advanced renal cell carcinoma or malignant melanoma. This study is comprised of two parts: Part 1, Dose Escalation (which has completed the enrollment and treatment phases and in which some patients continue in long-term follow up) and Part 2, Patient Expansion, which was initiated with Amendment 5.

4.1.1 Part 1, Dose Escalation

In Part 1, Dose Escalation, sequential cohorts of patients will be enrolled (Table 4-1). Each cohort will be assigned to receive doses of 0.1 mg/kg, 0.3 mg/kg, 1 mg/kg, 3 mg/kg, 10 mg/kg, or 15 mg/kg of GC1008 (according to the dose-escalation rules). Each dose of GC1008 will be given IV in an outpatient cancer treatment clinic or hospital setting. Prior to and over a period of 28 days following administration of the initial dose, blood will be collected for PK and PD studies. At the same time, safety information will be collected. If no DLTs are noted after the first dose of GC1008, patients will continue with GC1008 treatments at their assigned dose-level for 3 additional administrations given every 14 days.

Three patients will initially be enrolled and treated at the starting dose of 0.1 mg/kg GC1008. The decision whether to expand a cohort to 6 patients or to treat the next cohort of 3 patients at the next dose-level (0.3 mg/kg, 1 mg/kg, 3 mg/kg, 10 mg/kg, or 15 mg/kg) will be based on the occurrence of first-dose DLTs and will follow the dose-escalation rules. Escalation rules will be applied 28 days after the final patient in a given cohort receives the first treatment with GC1008. The MTD or maximal safe dose will be determined using predefined criteria.

During the conduct of this study, patient screening and enrollment efforts may overlap. In the event that 2 patients are simultaneously (i.e., within 10 days) found to be eligible to become the third patient on a given cohort, Genzyme may elect to allow a fourth patient to be enrolled in the cohort. The dose escalation rules will apply.

Table 4-1: Dosing Cohorts, Part 1

Dosing Cohort	Number of Patients	Dose (GC1008 MG/KG) (single injection, IV)	Dosing Regimen
1	3 to 6	0.1	Initial dose followed by a 28-day observation period. Thereafter, every 14 days for 3 additional doses. MTD or highest dose cohort will enroll 6 patients.
2	3 to 6	0.3	
3	3 to 6	1	
4	3 to 6	3	
5	3 to 6	10	
6	3 to 6	15	

If the safety profile of this agent permits, patients who demonstrate evidence of SD or tumor response (CR or PR) by Day 140 (End-of-Treatment Visit) may be offered Extended Treatment with GC1008 at the same dose they previously received, the current safe dose (the highest dose in which 0 of 3 or ≤1 of 6 patients experienced a DLT) or, if known, at the MTD or maximal safe dose.

Following completion of GC1008 treatment, patients will be asked to continue into long-term follow-up. Patients eligible for long-term follow-up include all patients who received GC1008 and meet criteria as outlined below. Long-term follow-up visits will occur every 3 months for up to 2 years or until 1 of the criteria is met. If a response occurs during long-term follow-up, efficacy assessment(s) will be evaluated by the Investigator to document a confirmed response.

Genzyme will provide ongoing safety monitoring and a DMC, consisting of 2 oncologists, will review safety data on a regular basis. Safety will be monitored by AE assessments, physical examination, vital signs, and clinical laboratory tests. Cumulative safety data will be prepared for DMC review at least every 3 months to assess any safety signals or trends. Safety issues that arise out of such reviews may lead to modifications of the treatment program.

4.1.2 Part 2, Patient Expansion

Part 1 (Dose Escalation) and a review of safety data have been completed as of Amendment 5. Of 22 patients enrolled and treated, no DLTs were observed during the study. The highest dose intended for administration in Part 1 of the study was 15 mg/kg. This dose was

determined to be the maximum safe dose administered and was thus selected for further study. Part 2 (Patient Expansion) will provide additional data regarding the safety of this 4 dose regimen, help assess the frequency of skin lesions and their relationship to any clinical response, provide additional PK, PD, and help define the dose levels, schedule, and dose modification guidelines that may be examined in a separate Phase 2 study in melanoma.

Two cohorts of 6 patients *may* be enrolled sequentially *in Part 2*. The first cohort will receive GC1008 at 15 mg/kg. Following review of *all available* safety data from *Part 1 and* the first cohort of 6 patients at 15 mg/kg *in Part 2*, a second cohort of patients *may* be treated at either the same level or at the reduced dose of 10 mg/kg (as described below)*, unless Genzyme, in consultation with the DMC, determines that sufficient safety data have been obtained, thus eliminating the need for continued enrollment.*

The initial cohort of 6 patients *in Part 2,* will be enrolled and treated with GC1008 at 15 mg/kg in a similar manner as in Part 1. Each dose of GC1008 will be given IV in an outpatient cancer treatment clinic or hospital setting. Prior to and over a period of 28 days following administration of the initial dose, blood will be collected for PK and PD evaluation. Patients will then continue with GC1008 treatments at 15 mg/kg for 3 additional administrations given every 14 days. Blood will be collected prior to and following each subsequent dose of GC1008 and during the 84 days (i.e., 12 weeks) following the final dose for PK and PD evaluation. In addition, routine laboratory tests will be performed for safety monitoring during the study. Baseline dermatologic exams will be obtained and skin assessments will be performed at each follow-up exam.

Patients will be re-staged 1 month (Day 84) and 3 months (Day 140) following therapy. If the safety profile of this agent permits, patients who demonstrate evidence of SD or tumor response (CR or PR) by physical examination or radiographic study and/or improvement of symptoms by the time of the End-of-Treatment Visit may be offered Extended Treatment with GC1008 and receive up to 2 additional courses as long as the benefit is maintained.

Following completion of GC1008 treatment, patients will be asked to continue into long-term follow-up. Patients eligible for long-term follow-up include all patients who received GC1008 and meet criteria. Long-term follow-up visits will occur every 3 months for up to 2 years or until 1 of the criteria are met.

For all patients in Part 2, Patient Expansion, random biopsies of uninvolved skin will be obtained pre- and post-treatment with GC1008 for exploratory analyses. Any patient who develops treatment emergent skin lesions will undergo dermatologic evaluations. These evaluations may include skin biopsies and photo documentation. Samples of these biopsies will be made available for review by a central pathologist.

In order to assess the impact of this 4 dose regimen, patients in Part 2 may be replaced if fewer than 4 patients receive 4 doses of GC1008 and have not completed evaluations sufficient to assess safety. This decision will be made by the Sponsor in consultation with the DMC. However, patients experiencing skin changes who are unable to complete all doses because of the event would not be replaced, unless following a review of the case by the Sponsor in consultation with the DMC, it is determined that replacement of the patient is warranted.

Following completion of therapy of the final patient in the first cohort of patients *in Part 2, Patient Expansion, all available safety data will be reviewed by Genzyme and the DMC to determine whether or not sufficient data have been obtained to meet the study's safety objectives and if continued enrollment is required. If it is determined that the safety objectives have not been met, a second cohort of 6 patients may be treated according to the same schedule described above. The decision whether to treat the second cohort of 6 patients at 15 mg/kg or to reduce the dose to 10 mg/kg would be based on the overall safety profile and the occurrence of "non-tolerable" skin events.*

Dose modification for an individual patient in either the first or second cohorts of Part 2 will be allowed provided the treatment is first discussed and agreed upon with the Sponsor and follows the general guidelines provided below. Treatment delays will be allowed. However, delays of >56 days (8 weeks) between scheduled doses will result in patient withdrawal from study treatment. Replacement of patients with treatment delays of >56 days (8 weeks) will be managed as described below.

During the patient expansion, all SAEs will be reported to the Sponsor. Safety will be monitored continuously, and all safety data will be reviewed with the DMC on a quarterly basis. At any time, an emerging safety profile of concern may result in modification of the study.

4.2 Patient Assignment and Identification

This is an open-label study. Patients will be enrolled and assigned to a treatment cohort only after they provide written informed consent, complete all pre-treatment assessments, and meet all requirements of the inclusion and exclusion criteria. The dosing cohort, patient identification number, and patient weight will be recorded in the electronic case report form (eCRF) and on the Drug Accountability Log, and study treatment infusion will be prepared accordingly.

A detailed description of patient identification is provided in the Study Operations Manual. All patients who provide written informed consent will be documented in the Patient Screening Log.

5. PATIENT POPULATION AND SELECTION

All inclusion and exclusion criteria must be met and confirmed prior to enrollment. Unless specified, all laboratory normal ranges that are mentioned in inclusion and exclusion criteria refer to institutional criteria. Unless otherwise specified, all inclusion, exclusion and patient withdrawal criteria apply to both Part 1 and Part 2 of this Phase 1 study.

5.1 Inclusion Criteria

1. **In Part 1, Dose Escalation:** Patients with histologically confirmed, locally advanced and surgically inoperable or metastatic renal cell carcinoma or malignant melanoma are eligible.

   In Part 2, Patient Expansion: Patients with histologically confirmed, locally advanced and surgically inoperable or metastatic malignant melanoma are eligible.

   In both Parts 1 and 2: All patients must have failed ≥1 prior therapy and potential patients may not be eligible for curative intent treatment (e.g., potentially curative surgical resection or chemotherapy). Other qualifying therapies include any medical, surgical, radiation, or investigational approach used for potential therapeutic benefit (but not for diagnostic purposes) in patients with advanced disease.

   In addition, in Part 1, patients with renal cell carcinoma must have failed temsirolimus and either sorafenib or sunitinib as part of their prior therapies.

2. Age ≥18 years.
3. Eastern Cooperative Oncology Group (ECOG) Performance Status 0 to 2.
4. Serum albumin ≥3.0 g/dL.
5. Expected survival ≥5 months.
6. Adequate organ function including:
   a. Marrow: Hemoglobin ≥10.0 g/dL, absolute neutrophil count (ANC) ≥1,500/mm^3, and platelets ≥100,000/mm^3.
   b. Hepatic: Serum total bilirubin ≤1.5 × upper limit of normal (ULN) (Patients with Gilbert's Disease may be included if their total bilirubin is ≤3.0 mg/dL), alanine aminotransferase (ALT), and aspartate aminotransferase (AST) ≤2.5 × ULN. If the patient has known liver metastases, an ALT and/or AST ≤5 × ULN are allowed.
   c. Renal: If negative proteinuria on urine dipstick, serum creatinine (sCR) <2 mg/dL or urine creatinine clearance ≥60 mL/min. If urine is 1+ positive (30 mg/dL), urine protein must be ≤1 g/24 hours and measured creatinine clearance ≥60 mL/min.
   d. Other: Prothrombin time (PT) and partial thromboplastin time (PTT) within normal ranges.

7. Measurable disease. Patients must have measurable disease as defined by Response Evaluation Criteria in Solid Tumors (RECIST) within 4 weeks prior to first dose of GC1008 (tumor lesion must be new or progressive if in a previously irradiated region).
8. Patients must have negative tests (antibody and/or antigen) for hepatitis viruses B and C and human immunodeficiency virus (HIV), unless the result is consistent with prior vaccination or prior infection with full recovery.
9. At the time of enrollment, patients must be >4 weeks since major surgery, radiotherapy, chemotherapy (≥6 weeks if they were treated with a nitrosourea, mitomycin, or monoclonal antibodies such as bevacizumab), immunotherapy, or biotherapy/targeted therapies and recovered from the toxicity of prior treatment to ≤ Grade 1, exclusive of alopecia. Concurrent cancer therapy is not permitted. (In patients who received long-acting agents, a treatment-free interval of 2 half-lives should be considered.)
10. Patients must be able to give written informed consent to participate. Patients may not be consented by a durable power of attorney.
11. Male and female patients of child-producing potential must agree to use effective contraception while enrolled on study and receiving the experimental drug, and for at least 3 months after the last treatment. Female patients of child-producing potential must have a negative serum pregnancy test confirmed within 7 days of receiving the initial dose of GC1008 therapy.
12. Documentation of flu vaccination if enrolled during flu season (as defined by the availability of vaccine). Otherwise, patients should receive the current flu vaccine ≥1 week before beginning GC1008 therapy.
13. Pre-treatment tumor samples, such as paraffin blocks or unstained slides, must be available for analyses.

5.2 Exclusion Criteria

1. Central nervous system (CNS) metastases, meningeal carcinomatosis, malignant seizures, or a disease that either causes or threatens neurologic compromise (e.g., unstable vertebral metastases).
2. History of ascites or pleural effusions, unless successfully treated, completely resolved, and the patient has not been treated for these conditions for >4 months.
3. Active thrombophlebitis, thromboembolism, hypercoagulability states, bleeding, or use of anti-coagulation therapy (including anti-platelet agents). Patients with a history of deep venous thrombosis may participate if successfully treated, completely resolved, and no treatment has been given for >4 months.

4. Hypercalcemia: Calcium >11.0 mg/dL (2.75 mmol/L) unresponsive or uncontrolled in response to standard therapy (e.g., bisphosphonates).
5. Pregnant or nursing women, due to the unknown effects of GC1008 on the developing fetus or newborn infant.
6. Patients diagnosed with another malignancy – unless following curative intent therapy, the patient has been disease free for at least 5 years and the probability of recurrence of the prior malignancy is <5%. Patients with curatively treated early-stage squamous cell carcinoma of the skin, basal cell carcinoma of the skin, or cervical intraepithelial neoplasia (CIN) are eligible for this study.
7. Patients with an organ transplant, including those that have received an allogeneic bone marrow transplant.
8. Use of investigational agents within 4 weeks prior to study enrollment (within 6 weeks if the treatment was with a long-acting agent such as a monoclonal antibody).
9. Patients on immunosuppressive therapy including:
   a. Systemic corticosteroid therapy for any reason, including replacement therapy for hypoadrenalism. Patients receiving inhaled or topical corticosteroids may participate.
   b. Patients receiving cyclosporine A, tacrolimus, or sirolimus are not eligible for this study.
10. Significant or uncontrolled medical illness, such as congestive heart failure (CHF), myocardial infarction, symptomatic coronary artery disease, significant ventricular arrhythmias within the last 6 months, or significant pulmonary dysfunction. Patients with a remote history of asthma or active mild asthma may participate.
11. Active infection, including unexplained fever (temperature >38.1°C), or antibiotic therapy within 1 week prior to enrollment.
12. Systemic autoimmune disease (e.g., systemic lupus erythematosus, active rheumatoid arthritis, etc.).
13. A known allergy to any component of GC1008.
14. Patients who, in the opinion of the Investigator, have significant medical or psychosocial problems that warrant exclusion. Examples of significant problems include, but are not limited to:
   a. Other serious non-malignancy-associated medical conditions that may be expected to limit life expectancy to less than 2 years (e.g., liver cirrhosis) or significantly increase the risk of SAEs.
   b. Any condition, psychiatric or otherwise, that would preclude informed consent, consistent follow-up, or compliance with any aspect of the study (e.g., untreated schizophrenia or other significant cognitive impairment).
   c. Patients currently abusing drugs or alcohol or, in the opinion of the Investigator, at high risk for poor compliance.

15. **Part 2 only**: Prior therapy with a TGFβ antagonist, such as an antibody, receptor, or kinase inhibitor or anti-sense therapy.

5.3 Patient Withdrawal

Patients are free to withdraw consent and discontinue participation in the study at any time, without prejudice to further treatment. A patient's participation in the study may also be discontinued at any time at the discretion of the Investigator or Sponsor. The following are reasons why the Investigator or Sponsor may remove a patient from study treatment and further follow-up:

- The patient withdraws consent;
- The patient is found to be not eligible after enrollment;
- The patient is non-compliant with study requirements;
- The Sponsor terminates the study.

The following are reasons why the Investigator or Sponsor may remove a patient from study treatment but continue follow- up:

- The patient becomes pregnant during the study;
- The patient suffers an intolerable AE. (An intolerable AE is an event which, even with optimal treatment, causes such discomfort or disability that the patient is unwilling to continue further study participation.);
- The patient experiences a treatment delay of >14 days in Part 1 or >56 days (8 weeks) between planned treatments in Part 2;
- The patient develops progressive disease;
- The patient requires a prohibited medication;
- For patients who develop study drug related skin lesions in Part 2:
    - Patient experiences intolerable symptoms despite treatment of symptoms, treatment break, and dose reduction;
    - Patient develops multiple (>5) treatment-emergent skin cancers that are proven by biopsy and central pathology review to represent cancers that clearly do not have features representative of keratoacanthoma or “keratoacanthoma-like” SCC or melanoma;
    - There is a significant change in risk/benefit, such as no evidence of reversibility of skin events following 8 weeks of observation off treatment; or
    - Use of systemic steroids for treatment of skin events.
- The patient experiences a DLT in Part 1 (see below);

Patients removed from study treatment will continue to be followed for safety and clinical outcome unless consent is withdrawn, subsequent cancer treatment is given, or there is disease progression and recommended alternative cancer treatment is declined.

In general, a patient experiencing a DLT in Part 1 will not receive additional GC1008 and will be withdrawn from the study. However, if upon review, Genzyme, the PI, and the DMC find that the study drug could continue with a reasonable margin of safety, treatment may be reinitiated at the same dose-level and the patient can be allowed to complete the course of GC1008 and continue on-study.

If a patient withdraws from the study after receiving GC1008, in either Part 1 or Part 2, the investigator should make every effort to perform a final assessment using the procedures set forth in the End-of-Treatment Visit , except as noted in Table 5-1. A final assessment should be performed within 30 days after withdrawal. Patients who withdraw from the study will be followed for new AEs and SAEs for 45 days after their last dose of GC1008, as outlined above. After the 45-day follow-up period, any SAE that comes to the attention of the site staff that may be causally related to GC1008 (i.e., there is a reasonable possibility that the event may have been caused by the drug) should be reported to Genzyme.

Table 5-1: Exceptions to End-of-Treatment Procedures

	Time of Patient Withdrawal	
**Procedure**	**Patient Withdraws Prior To Study Day 140 (Excluding Withdrawal During Extended Treatment)**	**Patient Withdraws During Extended Treatment**
Restaging CT (or MRI)	Not done if already performed within 30 days prior to End-of-Treatment Visit	Not done if already performed within 30 days prior to End-of-Treatment Visit
Restaging Bone Scan	Perform if patient had 1 bone scan completed on study, ≥2 months prior to visit	Perform if either of the following is true:<br>1) patient had ≤2 bone scans on study, with last scan ≥2 months prior to visit or<br>2) patient had >2 bone scans on study, with last scan ≥5 months prior to visit
PK Blood Collection	No exception – perform per protocol	No exception – perform per protocol

A patient who experiences a delay of >14 days in Part 1 or >56 days (8 weeks) between planned doses of GC1008 in Part 2 will be removed from study treatment. Under specific circumstances, Genzyme may choose to replace such patients.

There may be circumstances in which a patient who received GC1008 discontinues study participation before completing all scheduled treatments or evaluations. Since these patients may have experienced a response or benefit from treatment, they will be asked to continue study follow-up as outlined below.

In both Part 1 and Part 2, patients who discontinue study participation after receiving GC1008 but prior to the End-of-Treatment Visit (Day 140) or End-of-Extended Treatment Evaluation for any reason other than withdrawal of consent will be followed using the schedule set forth in the protocol. Patients will be followed every 3 months for up to 2 years after study discontinuation, until 1 of the following criteria is met:

- subsequent cancer treatment is given;
- disease progression and recommended alternative cancer treatment is declined; or
- death.

6. CLINICAL TRIAL MATERIAL ADMINISTRATION

6.1 Treatments Administered

GC1008 will be administered as IV infusions. It is suggested that patients be premedicated with diphenhydramine (e.g., 25 to 50 mg orally [PO] or IV) and acetaminophen (e.g., 500 to 650 mg PO) 30 to 90 minutes prior to treatment with GC1008. If diphenhydramine and acetaminophen are ineffective, ibuprofen (e.g., 600 mg PO) may be used.

The first dose of GC1008 will be administered IV in a stepwise manner in which the rate of infusion is incrementally increased. The rate of infusion and drug administration will vary, depending on the patient's weight and the dose administered. Please see the Study Pharmacy Manual for detailed information on administering GC1008.

In Part 1, Dose Escalation, patients will be assigned to receive GC1008 at 0.1 mg/kg, 0.3 mg/kg, 1 mg/kg, 3 mg/kg, 10 mg/kg, or 15 mg/kg (according to the dose-escalation rules). Patients will be observed and have vital signs monitored for 2 hours after completion of GC1008 administration or until discharge, whichever occurs later. The observation period

for subsequent infusions may be shortened to 1 hour for patients who did not experience an infusion-related reaction during the first 2 infusions. In all other circumstances, a 2-hour observation period will be required following later infusions.

During Part 1, Dose Escalation, patients will receive their initial dose and then be observed for 28 days for safety. PK and PD samples will be collected during this period. If no DLT occurs, each patient may continue treatment and receive 3 additional treatments, given 14 days apart.

In Part 2, Patient Expansion, patients will receive GC1008 at 15 mg/kg or 10 mg/kg. Six patients in the first cohort will be treated with 15 mg/kg. Following *completion* of the first cohort of patients at 15 mg/kg *in Part 2, a second cohort of six patients may be treated at 15 mg/kg or the reduced dose of 10 mg/kg, unless Genzyme, in consultation with the DMC, determines that sufficient safety data have been obtained to meet the study's safety objectives, thus eliminating the need for continued enrollment. If the safety objectives have not been met*, a second cohort of 6 patients will be treated *with GC1008, and a*dministration of *study drug* will be identical to that described above except that patients will be observed and their vital signs will be monitored for 1 hour after completion of GC1008 administration or until discharge, whichever occurs later. After receiving the initial dose, patients will have PK and PD samples collected over the next 28 days to determine the effects of a single dose of GC1008. Patients will then receive 3 additional treatments, given 14 days apart. Additional PK and PD samples will be collected during and following these treatments to determine the effects of multiple doses of GC1008.

In both Parts 1 and 2, any patient treated with GC1008 who develops ≥ Grade 2 acute infusion adverse reactions will be monitored for an additional 1 to 2 hours beyond standard post-infusion monitoring or until the AE resolves to < Grade 2. Medically indicated treatment of the AE will be administered. If an acute AE of Grade 2 or greater persists for >4 hours during observation or despite treatment, a decision to continue observation, institute or modify treatment of the AE, or admit the patient to an inpatient unit for further observation and/or treatment will be made by the Investigator. The case will then be reviewed by the Investigator and Genzyme to determine if administration of GC1008 may continue in this patient.

The following applies to AEs observed in both Parts 1 and 2. Skin events will follow guidelines outlined in the protocol. Prior to each subsequent dose of GC1008 (i.e., doses 2, 3, and 4), each patient will be evaluated for the development of AEs. Any patient experiencing an AE that is considered related to study drug (possibly, probably, or definitely related) must have recovered to ≤ Grade 1 or baseline prior to receiving the next dose of GC1008. Any patient experiencing an AE that is considered unrelated to study drug (such as those clearly related to underlying cancer or pre-existing conditions), which has not recovered to ≤ Grade 1 or baseline, may continue GC1008 if the Investigator and Genzyme agree that continuing study drug is not likely to pose a significant safety risk. Treatment delays of >14 days for Part 1 and >56 days (8 weeks) between planned treatments for Part 2 **will** result in withdrawal of the patient from study treatment.

If an identical or similar study drug related AE reoccurs after further GC1008 administration and both events are Grade 2 or 3, the events must be reviewed by Genzyme and the DMC before additional treatment with GC1008 will be allowed. If Genzyme and the DMC determine that a significant safety risk exists, the patient will be withdrawn from the study. If an identical or similar study drug related AE reoccurs after further GC1008 administration and either event is Grade 4, the patient will be withdrawn from the study.

After completing all 4 doses of GC1008 in Part 1 or 2, if the patient is found to have SD or evidence of tumor response (CR or PR) by physical examination or radiographic study and/or improvement of symptoms by the time of the End of Treatment visit, they may be offered Extended Treatment with GC1008 and receive up to 2 additional courses as long as the benefit is maintained.

6.1.1 Dosage

The dose of GC1008 administered will be based on the patient's actual body weight (ABW) as measured on Day 0. Subsequent doses will be recalculated if the patient's weight changes by ≥10%.

6.2 Investigational Product

GC1008 is an engineered human monoclonal antibody against human TGFβ1, β2, and β3

6.2.1 Dose-Escalation Rules (Part 1)

1. If 0 first-dose DLTs are observed out of 3 (or 4) patients treated with a dose-level, the next cohort of 3 (or 4) patients may be enrolled and treated at the next dose-level.
2. If 1 out of the 3 (or 4) patients treated with a dose-level experience a first-dose DLT, up to 3 additional patients (6 total) will be enrolled at that dose-level.
   a. If only 1 out of these 6 patients experiences a first-dose DLT, then the next cohort of 3 (or 4) patients may be enrolled and treated at the next dose-level.
   b. If ≥2 out of these 6 patients experience first-dose DLTs, then dose escalation is stopped. Additional patients will be enrolled at the prior lower dose-level to achieve a total of 6 patients.
3. If ≥2 out of 3 (or 4) patients in a cohort experience a first-dose DLT, dose escalation will be stopped. Additional patients will be enrolled at the prior lower dose-level, to achieve a total of 6 patients.
4. If 0 or 1 out of 3 (or 4) patients at the highest dose-level (15 mg/kg) experiences a DLT, additional patients will be enrolled to achieve a total of 6 patients.
   a. If ≤1 out of these 6 patients experiences a DLT, the 15 mg/kg dose-level will be declared the maximal safe dose administered in this Phase 1 study.
   b. If ≥2 patients experience a DLT, additional patients will be enrolled at the prior lower dose-level, for a total of 6 patients.

Escalation rules will be applied 28 days after the final patient in a given cohort receives the first treatment with GC1008. If ≤1 out of 6 patients at the highest dose-level (15 mg/kg) experiences a DLT, this dose-level will be declared the maximal safe dose used in this study.

If 15 mg/kg is not found to be safe, the MTD is defined as the next highest dose-level in which ≤1 out of 6 patients experiences a first-dose DLT.

6.2.2 Guidelines for Treatment Delay, Dose Reduction, and Discontinuation (Part 2)

Patients experiencing symptoms associated with skin events or AEs that in the opinion of the investigator require a treatment delay or dose modification should be discussed with the Sponsor. Treatment delays are allowed but if the time interval between planned doses is >56 days (8 weeks), this will result in discontinuation of study treatment for the patient. (The only exception to this would be delays that are considered non-safety related or related to the evaluation of the event such as a delayed central pathology review).

6.2.2.1 Treatment Delays (Part 2 Only)

1. Treatment Delays
   a. Treatment must be delayed after initial presentation of skin lesions until biopsies are obtained and the results are known. The type of biopsy performed is at the discretion of the site; however, it is recommended that a full thickness incisional or punch biopsy be performed to best evaluate these lesions. If multiple lesions occur, several lesions should be removed for purposes of sampling; the exact number of biopsies is at the discretion of the site. Treatment decisions may be made following site or local pathology review. All samples will be collected and reviewed by a central pathologist acting as consultant for Genzyme.
   b. Treatment may be delayed at the Investigator's discretion if the patient is symptomatic
2. Re-treatment of patients whose therapy was delayed for symptoms
   a. After a treatment delay with a minimum of 28 days (4 weeks) between planned doses, if symptoms improve, treatment may be restarted at the same dose at the Investigator's discretion. (Planned doses are administered at scheduled intervals of 2 or 4 weeks. If the interval between planned doses is 2 weeks, an additional 2 week delay is mandatory. If the interval between planned doses is 4 weeks, then no additional delay is required if symptoms improve.)

6.2.2.2 Dose Reduction (Part 2 Only)

In Part 2, all dose reductions should first be discussed between the Investigator and Sponsor. (No dose reductions were allowed in Part 1.)

If a patient in Part 2 develops worsening symptoms associated with skin events following re-treatment at the same dose level that are grade 3/4 (with the exception of new grade 3 skin cancers) or are intolerable, the dose may be dropped one dose level following a treatment break where symptoms improve to ≤ grade 2 and become tolerable. For patients initially receiving GC1008 at 15 mg/kg, the dose will be reduced to 10 mg/kg, and for patients initially receiving GC1008 at 10 mg/kg, the dose will be reduced to 3 mg/kg. Only one dose level reduction is allowed per treatment course.

6.2.2.3 Discontinuation of Treatment Due to Skin Events (Part 2 Only)

All discontinuations should first be discussed between the Investigator and Sponsor prior to withdrawal of treatment. Treatment should be discontinued if:

- Patient experiences intolerable symptoms despite treatment of symptoms, treatment break and dose reduction,

- Patient develops multiple (>5) treatment emergent, skin cancers which are proven by biopsy and central pathology review to represent cancers that clearly do not have features representative of keratoacanthoma or “keratoacanthoma-like” SCC or melanoma
- There is a significant change in risk/benefit, such as no evidence of reversibility following 8 weeks of observation off treatment
- Use of systemic steroids or other prohibited medications is required to treat the skin lesions

7. EFFICACY AND SAFETY VARIABLES

7.1 Efficacy and Safety Measurements Assessed and Study Flowchart

The study will be conducted as outlined in the following sections, unless otherwise specified all assessments will be performed in both Parts 1 and 2 of the study. Study procedures and assessments are presented by visit for Parts 1 and 2 in Table 7-1 and for additional procedures conducted during Part 2 only in Table 7-2. At each visit, the site should confirm that patients with child-producing potential continue to practice effective contraception.

Response evaluations will be performed at 1 and 3 months following the planned 4 treatments. Whenever an objective response is noted, confirmatory scans or other appropriate studies must be obtained at least 4 weeks after the response is first documented. Study visits will be based on calendar days from Day 0.

Table 7-1: Schedule of Study Events For Parts 1 and 2

	Screening and Baseline		Day 0	Day 1	Day 3	Day 7	Day 14	Day 28	Day 42	Day 56
	Day -28 to -1	Day -7 to -1								
Written Consent	X									
Medical History and Baseline Characteristics	X									
Physical Examination and Oral Assessment		X					X	X	X	X
Weight			X					X	X	X
Height			X							
ECOG Performance Status	X	X					X	X	X	X
Vital Signs		X	X				X	X	X	X
Clinical Laboratory Tests[1]	X	X	X	X	X	X	X	X	X	X
PT and PTT Tests	X	X	X	X		X		X	X	X
Albumin and Magnesium		X	X							
Hepatitis B, Hepatitis C, and HIV Testing[2]	X									
Electrocardiogram	X									
Flu Vaccine[3]	X									
CT Scan of Chest, Abdomen, and Pelvis; Assessment of Tumor Measurement by Radiographic and Other Methods (Use PET CT scans[4] of chest, abdomen and pelvis for dose cohorts 3, 10, and 15 mg/kg)	X									
Head CT Scan with/without contrast, or MRI	X									
Assessment of Tumor Measurement by Physical Examination	X	X					X	X	X	X
Occult Blood Test and Urine Protein	X									
Obtain Tumor Blocks or Slides	X									

Footnotes appear on the last page of Table 9-1.

Table 9-1: Schedule of Study Events For Parts 1 and 2 (continued)

	Screening and Baseline		Day 0	Day 1	Day 3	Day 7	Day 14	Day 28	Day 42	Day 56
	Day -28 to -1	Day -7 to -1								
Bone Scan	X									
Bleeding Time[5]		X								
Serum Pregnancy Test		X								
Obtain Enrollment Authorization		X								
Blood GC1008 PK Assay			X	X	X	X	X	X	X	X
Blood GC1008 Antibody Assay			X					X		
GC1008 Infusion			X					X	X	X
SAE Assessment	X	X	X				X	X	X	X
AE Assessment (starting with enrollment)		X	X				X	X	X	X
Concomitant Medication/Therapy (starting with enrollment)		X	X				X	X	X	X

Footnotes appear on the last page of Table 9-1.

Table 9-1: Schedule of Study Events For Parts 1 and 2 (continued)

	Day 63	Day 70	Day 84	Day 112	Day 140 End-of-Treatment Visit[6]	Long-Term Follow-up[7] Every 3 Months
Physical Examination and Oral Assessment			X		X	
Weight						
ECOG Performance Status			X		X	X
Vital Signs			X		X	
Clinical Laboratory Tests[1]	X		X	X	X	
PT and PTT Tests	X					
CT Scan of Chest, Abdomen, and Pelvis; Assessment of Tumor Measurement by Radiographic and Other Methods (Use PET CT scans of chest, abdomen and pelvis for dose cohorts 3, 10, and 15 mg/kg)			X		X	X[8]
Assessment of Tumor Measurement by Physical Examination			X		X	X
Bone Scan			X		X	
Bone Scan (performed if applicable)						X
Bleeding Time Test[5]	X					
Blood GC1008 PK Assay	X	X	X	X	X	X[9]
Blood GC1008 Antibody Assay			X		X	X[9]
SAE Assessment			X	X	X	X[10]
AE Assessment			X	X	X	
Concomitant Medication/Therapy			X	X	X	
Additional Cancer Therapy						X
Footnotes appear on the last page of Table 9-1.						

Table 9-1: Schedule of Study Events For Parts 1 and 2 (continued)

Table footnotes:

[1] Clinical laboratory tests include creatinine, calcium, phosphate, AST, ALT, total and direct bilirubin, LDH, alkaline phosphatase, BUN, sodium, potassium, chloride, bicarbonate, and complete blood count (CBC) with differential and platelets.

[2] HIV testing must be performed within 3 months of patient consent.

[3] Flu vaccine will be administered on Day -7 or earlier if the patient is enrolled during flu season.

[4] PET CT scans alone may be used if normal resolution CT scans with contrast are performed as part of the study so that tumor measurements are accurate. Otherwise, normal resolution CT scans (with and without contrast) of the chest, abdomen, and pelvis must be performed in addition to the PET study.

[5] If unavailable, platelet function tests (platelet aggregation [performed by standard or automated tests such as the PFA-100] and von Willebrand's factor) may substitute.

[6] If the patient withdraws from the study, the patient must be followed for new AEs and SAEs for 45 days after the last dose.

[7] These assessments are performed if the patient has not received alternative cancer treatment or has disease progression but has not declined recommended alternative cancer treatment.

[8] PET CT scans are not required during Long-Term Follow-up.

[9] Additional samples for anti-GC1008 antibody tests and PK may be requested during long-term follow-up if needed to complete the analyses.

[10] If SAEs come to the attention of the site staff following the End-of-Treatment Visit, they will be reported to Genzyme if they may be causally related to study drug.

Table 7-2: Schedule of Study Events For Additional Procedures Conducted During Part 2 Only

	Screening and Baseline							Day 140 End-of-Treatment Visit[2]
	Day -28 to -1	Day -7 to -1	Day 14	Day 28	Day 42	Day 56	Day 84	
Skin Assessment[1]		X	X	X	X	X	X	X
Complete Dermatologic Assessment	X							
Biopsy of uninvolved skin	X						X	

1 During Part 2, the physical examination will include a skin assessment.
2 If the patient withdraws from the study, the patient must be followed for new AEs and SAEs for 45 days after the last dose.

Table 7-3: Schedule of Study Events During Extended Treatment – Parts 1 and 2

	End-of-Initial Treatment[1]	Day 0	Day 14	Day 28	Day 42	Day 49	Day 70[4]
Physical Examination and Oral Assessment	X	X	X	X	X	X	X
Vital Signs	X	X	X	X	X	X	X
Weight		X	X	X	X		
ECOG Performance Status	X	X	X	X	X	X	X
Clinical Laboratory Tests[2]	X	X	X	X	X	X	X
PT and PTT Tests		X					
Albumin and Magnesium		X					
Assessment of Tumor Measurement by Physical Examination	X	X	X	X	X	X	X
CT Scan of Chest, Abdomen, and Pelvis; Assessment of Tumor Measurement by Radiographic and/or Other Methods[3]	X						X
Blood GC1008 PK Assay[4]	X						
Blood GC1008 Antibody Assay[4]	X						X
GC1008 Infusion		X	X	X	X	6-week rest after 4th dose	
SAE and AE Assessment	X	X	X	X	X	X	X
Concomitant Medication/Therapy	X	X	X	X	X	X	X
Assess for Continuation of Extended Treatment	X					X	X

1 The End-of-Initial Treatment Visit may be combined with another scheduled study visit, but evaluations must be completed within 35 days prior to Day 0 of Extended Treatment.

2 Clinical laboratory tests include creatinine, calcium, phosphate, AST, ALT, total and direct bilirubin, CBC with differential, platelets, LDH, alkaline phosphatase, BUN, sodium, potassium, chloride, and bicarbonate.

3 Restage the patient using CT or MRI scans (with and without contrast) of the chest, abdomen, and pelvis.

4 After completing the final course of Extended Treatment, patients will undergo an assessment using the procedures outlined in the End-of-Treatment Visit; this visit will substitute for the Extended Treatment Day 70 visit. **For patients in Part 2**, additional PK and anti-GC1008 samples may be requested to complete analyses.

Table 7-4: Schedule of Study Events During Extended Treatment – Part 2 Only

	End-of-Initial Treatment[1]	Day 0	Day 14	Day 28	Day 42	Day 49	Day 70
Skin Assessment[2]		X	X	X	X	X	X
Blood GC1008 PK Assay		X	X	X	X	X	X
Blood GC1008 Antibody Assay							X

[1] The End-of-Initial Treatment Visit may be combined with another scheduled study visit, but evaluations must be completed within 35 days prior to Day 0 of Extended Treatment.

[2] During Part 2, the physical examination will include a skin assessment.

7.2 Adverse Events

At each study visit, patients will be evaluated for new or evolving AEs. The physician should elicit symptoms using an open-ended question, followed by appropriate questions that clarify the patient's verbatim description of AEs or change in concomitant medications. A review of systems will be performed.

7.2.1 Part 1: Dose-Limiting Toxicity

A DLT is defined as any of the AEs defined below that occur within the first 28 days following the initial treatment with GC1008:

1. acute infusion reaction/cytokine release syndrome of ≥ Grade 3 despite maximal appropriate therapy and allowing ≥4 hours for that therapy to take effect,
2. any hematological toxicity ≥ Grade 3 that is considered causally related to GC1008, with the following exceptions:
   a. Neutropenia will be considered dose-limiting only when the ANC is <1,000/mm^3 for >96 hours (4 days) or when it is associated with fever.
   b. Thrombocytopenia will be considered dose-limiting only if platelets are <50,000/mm^3 for >96 hours (4 days), or
3. any ≥ Grade 3 non-hematologic toxicity that is considered causally related to GC1008, with the following exceptions:
   a. ≥ Grade 3 diarrhea or vomiting will constitute a DLT only if it occurs despite treatment with optimal anti-diarrheals or anti-emetics.
   b. If a patient with known liver metastases was enrolled with Grade 2 AST or ALT laboratory abnormalities at baseline, an increase in AST or ALT will be considered dose limiting only if the increase is >3 times the baseline and the elevation is confirmed ≥5 days later. (Note: the elevated values are Grade 3 by CTCAE criteria.)
   c. If a patient with Gilbert's Disease was enrolled with a Grade 2 bilirubin abnormality at baseline, an increase in bilirubin will be considered dose limiting only if the increase is >3 times the baseline and the elevation is confirmed ≥5 days later.
   d. Fatigue will be considered dose limiting only if ≥ Grade 4.

There is no provision made in this protocol for reduction of the dose of the GC1008 treatment based on toxicity.

To expedite reporting, all DLTs will be collected using the SAE reporting mechanism.

Safety will be monitored and examined on a continuous basis and such reviews may lead to a modification of the treatment at any time.

7.2.2 Non-Tolerable Skin Events

Non-tolerable skin events are defined as AEs that would not be expected to be ameliorated by dose, dose schedule modifications, or other treatments. These events may include:

- Skin lesions that involve >50% of the body surface, are symptomatic (pruritus, pain, etc.), and do not improve following appropriate symptom treatment, treatment break, and dose reduction
- The appearance of multiple (>5) treatment emergent skin cancers which are proven by biopsy and central pathology review to represent cancers that clearly do not have features representative of keratoacanthoma or "keratoacanthoma-like" SCC or melanoma.
- "Other" events that in the opinion of the investigator and the Sponsor are considered "non-tolerable" and unlikely to be ameliorated by schedule or dose modification

8. STATISTICAL METHODS AND PLANNED ANALYSES

The Sponsor or designee will be responsible for:

- Patient listings
- Statistical analysis
- Combined clinical and statistical study report

All data recorded in eCRFs, as well as any outcomes derived from the data, will be presented in summary tables and/or data listings. The detailed analysis methods are included in the Statistical Analysis Plan.

In this study, patients may have more than 1 course of GC1008. A study report will be produced once all the patients in Parts 1 and 2 have either completed 1 course of GC1008 or discontinued from the study. An Addendum will follow upon completion of the study.

8.1 Demographics and Baseline Characteristics

Demographic and other background variables will be summarized. For categorical variables, frequencies and percentages will be presented. Continuous variables will be summarized by mean, median, standard deviation, and range.

8.2 Concomitant Medication Usage

Concomitant medication usage will be presented in individual patient listings.

8.3 Patient Accountability

The number of patients who enrolled into the study, the frequency and percentage of patients who withdrew from the study, and the reasons for withdrawal, will be tabulated.

8.4 Study Treatment Usage and Compliance

A summary of study treatment administration and compliance will be presented for all patients.

8.5 Efficacy Endpoints

Tumor response (progressive disease, SD, PR, or CR) will be assessed by RECIST criteria, as discussed in Appendix B. Efficacy assessments will be made by the Investigator at the

investigational site. If necessary, Genzyme may request films and other components of response assessments for review.

Tumor response is being explored in this Phase 1 study. Patient data will be analyzed on the basis of tumor type and dose administered. The proportion of patients with CR; with PR or better; and with SD or better will be presented, along with the corresponding 90% confidence interval. The duration of responses will be examined, and progression-free survival will be estimated using the Kaplan-Meier method.

8.5.1 Intent-to-Treat (ITT) Population

The Intent-to-Treat (ITT) population includes all enrolled patients who are confirmed to be eligible for entry into the study and all treated patients, even if they are later determined to be ineligible. This set of patients will be referred to as the Full Analysis Set.

8.5.2 Evaluable-for-Response Population

The Evaluable-for-Response Population is a subset of the ITT population. Patients will be considered evaluable for purposes of assessing overall response rate and duration of response only if they have completed at least 1 treatment and have undergone at least 1 appropriate tumor response assessment. This set of patients will be referred to as the Per Protocol Set.

8.5.3 Safety Population

In both Part 1 and Part 2, all patients who sign informed consent and receive at least one dose of study drug will be included in the Safety population (Safety Set).

8.6 Safety Endpoints

The safety analyses will be performed on the Safety Set. Study-procedure-related SAEs (only) will be recorded for patients who sign informed consent and receive at least one dose of study drug. Safety analyses will include all data available at the time of database lock. After the last database lock, follow-up information on SAEs and newly reported related SAEs will be managed through the Pharmacovigilance database.

To investigate dose response, safety parameters will be tabulated by cohort. Patients who demonstrate evidence of tumor response (CR, PR, SD) by physical examination or radiographic study and/or improvement of symptoms may receive Extended Treatment,

which may bias the assessment of dose response. Exploratory analyses to examine the relationship between dose, duration of treatment, and safety parameters will be undertaken.

Safety assessments to be summarized are:

- AEs
- DLTs
- Changes in clinical laboratory assessments from pre-treatment to study visits
- Change in vital signs from pre-treatment to study visits
- Changes in physical examination from pre-treatment to study visits
- Evaluation of development of antibodies to GC1008

8.6.1 Adverse Events

AEs will be classified using a current version of the MedDRA coding dictionary. All reported AEs will be displayed in patient listings. The safety assessment will be based on the incidence of treatment-emergent AEs, including summaries of SAEs, treatment-limiting toxicities, AEs leading to withdrawal, and AEs deemed related to study treatment. The severity of each AE will be assessed using the NCI CTCAE Toxicity Scale, Version 3.0, dated 12 December 2003, Appendix C. If CTCAE does not apply, criteria in described above will be used.

AEs will be categorized and tabulated by system organ class, preferred term, intensity, seriousness, outcome, and relationship to study treatment. Additionally, discontinuations due to AEs, will be summarized. Information collected prior to GC1008 treatment will be presented separately from treatment-emergent signs and symptoms.

If a patient has more than 1 occurrence of an AE for a specific preferred term, the patient will be counted only once for that preferred term. The most intense occurrence of an AE, as well as the most extreme relationship of the AE to the study treatment, will be indicated in cases of multiple occurrences of the same AE.

Changes from pre-treatment in clinical laboratory tests will be summarized. The analysis of laboratory values will also be based on frequencies of abnormal values. Safety outcomes information will be handled by providing counts and percentages if categorical, and by

providing univariate descriptive statistics if continuous. Genzyme will provide cumulative data (as specified in the DMC charter) to the DMC for review.

8.6.2 Laboratory Parameters

Abnormal clinical laboratory values will be noted as either high or low based on the normal ranges for each laboratory parameter. Changes from pre-treatment in laboratory parameters will be summarized.

8.6.3 Other Safety Assessments

Listings of vital sign and physical examination data will be presented.

8.7 Maximum Tolerated Dose or Maximum Safe Dose

The MTD or maximum safe dose will be determined as outlined above. The tolerability of repeat dosing will be evaluated based on the safety profile.

8.8 Pharmacokinetic Endpoints

Pharmacokinetics of GC1008 will be assessed using noncompartmental methods using actual blood collection times. Areas will be calculated using the linear trapezoidal rule. The elimination rate constant will be estimated based on linear regression of at least 3 data points having a coefficient of determination greater than 0.90. Maximal concentrations and time to maximal concentrations will be determined using observed concentrations. If noncompartmental methods are inadequate to characterize the pharmacokinetics of GC1008, a compartmental approach under a nonlinear mixed-effects model paradigm will be used. The following primary endpoints will be calculated: clearance, volume of distribution, and in the case of multiexponential disposition profiles, volume of distribution at steady-state. The secondary parameters of interest are total area under the curve assuming a single dose from time 0 to infinity, maximal concentration, time to maximal concentration, and half-life.

All PK parameters will be tabulated by individual and summarized by descriptive statistics by cohort. Concentrations will be summarized using descriptive statistics based on nominal collection time. Individual plots will also be generated.

8.9 Anti-GC1008 Antibody Analysis

Serum samples will be collected at the time points specified in Table 7-1 and Table 7-3 and analyzed for the presence of antibodies directed against GC1008. Analyses will be performed using an ELISA-based assay. The presence and relative titer of such human anti-human antibody responses will be assessed and summarized for the patient population.

A total of 4 blood samples in Part 1 and 3 blood samples in Part 2 will be collected over a period of approximately 140 days, with a total blood volume of approximately 12 to 18 mL. One sample will be collected in each course of Extended Treatment (approximately 4.5 mL). Additional samples for anti-GC1008 antibody tests may be requested during long-term follow up, if needed to complete the analyses.

8.10 Statistical Analysis Plan

A statistical analysis plan will be prepared prior to database lock.

8.11 Power and Sample Size

The objective of this study is to assess the safety and PK profile of GC1008. In Part 1, Dose Escalation, the sample size of at least 3 patients in each cohort is the size typically used in a dose-escalation study.[1] Cohorts may be expanded if there is a need to gather additional information on safety. Up to 36 patients will be enrolled in Part 1, Dose Escalation. In Part 2, Patient Expansion, approximately 12 patients will be enrolled, with 2 cohorts of 6 patients each to be enrolled sequentially. The dose level in the second cohort will be determined based on the outcome of the safety review conducted on the initial cohort of 6 patients.

Because patients with 2 types of malignancies will be eligible for this study, the number of patients with a specific type of cancer is small, and meaningful statistics are unlikely. Nevertheless, patients with measurable disease will be assessed by standard criteria, and the objective tumor response rates will be examined. Estimates of response rates will be provided with 90% CIs. For a sample size of 24 and assuming a response rate of approximately 20%, we would expect to observe 4, 5, or 6 responses. Assuming 4 responders, the lower limit of the CI is 5.9%. Assuming 5 responders, the lower limit of the CI is 8.6%. Assuming 6 responders, the lower limit of the CI is 11.5%.

8.12 Other Statistical Issues

8.12.1 <u>Significance Levels</u>

CIs for tumor response will be based on an alpha of 0.10. All other analyses will use an alpha of 0.05.

8.12.2 <u>Missing or Invalid Data</u>

Missing or invalid data will not be replaced.

8.12.3 <u>Computing Environment</u>

All analyses will be performed using SAS® and WinNonlin (Pharsight Corporation).

9. REFERENCES

1 Simon RM. Design and analysis of clinical trials. In: DeVita VT, Hellman S, Rosenberg SA, eds. Cancer: Principals and Practice of Oncology. 6th ed. Philadelphia, PA: J.B. Lippincott Company, 2001.
