# Supplementary material for: Phase I Study of GC1008 (Fresolimumab): A Human Anti-Transforming Growth Factor-Beta (TGFβ) Monoclonal Antibody in Patients with Advanced Malignant Melanoma or Renal Cell Carcinoma
Source: PLoS One. 2014 Mar 11;9(3):e90353. doi: 10.1371/journal.pone.0090353 (PMC3949712; doi:10.1371/journal.pone.0090353)
Supplement: Table S1 — Individual Patient Data. (DOC) [file pone.0090353.s005.doc]

**Table 1 Supplemental: Individual Patient Data**

|  |  |  | | **Total (N=29)** | | | | | | |  | |
| --- | --- | --- | --- | --- | --- | --- | --- | --- | --- | --- | --- | --- |
| **Dose Cohort** | **Patient** | **Disease** | **Stage at Entry** | **TNM** | **Sites of Metastatic Disease** | **Prior Treatments** | | **Time from Diagnosis (months)** | | **GC1008 Treatment (# doses)** | | **Best Response** |
| 0.1 mg/kg |  |  |  |  |  |  | |  | |  | |  |
|  | 001 | MM | IV | T4aN3M1c | Liver, lung, abdomen, LN | B, C, S, V | | 224 | | 3 | | PD |
|  | 002 | MM | IV | TxN1bM1b | Lung, LN | S | | 93 | | 4 | | PD |
|  | 003 | MM | IV | T0N3M1c | Lung, LN | C, K, S | | 54 | | 4, ET 4(1mg/kg) | | SD |
| 0.3 mg/kg |  |  |  |  |  |  | |  | |  | |  |
|  | 004 | MM | IV | TxNxM1b | Lung, LN | C, S, R | | 24 | | 4 | | PD |
|  | 005 | MM | IV | TxN0M1c | Liver, lung, abdomen, adrenal, bone/spine, skin | B, C, S, V | | 47 | | 4 | | PD |
|  | 006 | MM | IV | T3aN1bM1b | Liver, lung, LN, skin | B, C, S, R | | 10 | | 4, ET 4(1mg/kg) | | SD |
| 1 mg/kg |  |  |  |  |  |  | |  | |  | |  |
|  | 007 | MM | IV | T0NxM1a | Skin, probable LN | B, C, S | | 339 | | 4, ET 4(3mg/kg), 4(15mg/kg) | | PR |
|  | 008 | MM | IV | T0N0M1c | Liver, lung | B. S, V | | 206 | | 4, ET 4(3mg/kg) | | SD |
|  | 009 | MM | IV | T0N0M1a | Lung, skin, peri-renal | C, K, S, R | | 24 | | 4, ET 4(3mg/kg) | | SD |
| 3 mg/kg |  |  |  |  |  |  | |  | |  | |  |
|  | 010 | MM | IV | T0N1bM1c | Adrenal, LN, infraclavicular mass | B, S | | 107 | | 4 | | PD |
|  | 011 | MM | IV | TxN1bM1a | Pelvis, LN | C, S | | 50 | | 4 | | PD |
|  | 012 | MM | IV | TxNxM1c | Liver, Lung, pancreas, breast, bone/spine | S | | 368 | | 4 | | SD |
|  | 013 | MM | IV | M1a | Lung, peri-pancreatic, LN | B, S | | 21 | | 3 | | PD |
| 10 mg/kg |  |  |  |  |  |  | |  | |  | |  |
|  | 014 | MM | IV | TxNxM1c | Adrenal, liver, spleen, LN | B, S | | 33 | | 3 | | PD |
|  | 015 | MM | IV | TxN2aM1c | Liver, lung, neck, LN | B, C, S, V | | 62 | | 1 | | PD |
|  | 016 | RCC | IV | T3bN0M1 | Renal, lung, liver, cardiac, skin | B, K, S | | 20 | | 4 | | PD |
| 15 mg/kg |  |  |  |  |  |  | |  | |  | |  |
|  | 017 | MM | IV | TxNxM1c | Skin, LN | B, S, V, R | | 14 | | 2 | | PD |
|  | 018 | MM | IV | T4aN2aM1a | Skin, LN | B, C, S, V | | 205 | | 4 | | SD |
|  | 019 | MM | IV | T3aNxM1c | Liver, lung, spleen, skin | C, S | | 375 | | 3 | | NA |
|  | 020 | MM | IV | TxN3M1b | Lung, LN, skin | B, C, K, S | | 23 | | 3 | | PD |
|  | 021 | MM | IV | T4bNxM1c | Peritoneal mass, abdomen, muscle, peri-nephric, prostate, spleen | B, S | | 116 | | 1 | | PD |
|  | 022 | MM | IV | T2aN3M1b | Lung, LN, skin | B, C, S | | 20 | | 4 | | PD |
|  | 023 | MM | IV | T4bN3M1b | Lung, LN, skin | B, C, S, V | | 50 | | 4 | | PD |
|  | 024 | MM | IV | TxN3M1c | Liver, lung, adrenal, LN, skin | B, C, K, S, V | | 56 | | 3 | | PD |
|  | 025 | MM | IV | T0N0M1b | Ileum, lung | C, K, S, R | | 65 | | 3 | | PD |
|  | 026 | MM | IV | T4aN2aM1c | Pelvis, LN | B, C, K, S | | 59 | | 4 | | PD |
|  | 027 | MM | IV | TxNxM1a | LN | B, S | | 107 | | 2 | | PD |
|  | 028 | MM | IV | T4aN3M1b | Lung, skin | B, S, R | | 26 | | 4 | | PD |
|  | 029 | MM | IV | TxNxM1b | Lung, LN, skin | B, S | | 9 | | 1 | | PD |
|  |  |  |  |  |  |  |  | |  | |  | |
|  |  |  |  |  |  |  |  | |  | |  | |
|  |  |  |  |  |  |  |  | |  | |  | |

Four patients were enrolled into the 3mg/kg cohort. As a multi-center study, it was recognized that patient screening and enrollment efforts could overlap. In the event that 2 patients were simultaneously (i.e., within 10 days) found to be eligible to become the third patient on a given cohort, the protocol specifically allowed a fourth patient to be enrolled in the cohort.

Skin: includes subcutaneous nodules/masses

C: chemotherapy

B: biologic

S: surgery

V: vaccine

K: kinase inhibitor (sorafenib, sunitinib, dasatinib, gamma secretase inhibitor, not specified)

R: radiation therapy

ET: Extended Therapy
